# Supplementary material for: Derivation and utility of schizophrenia polygenic risk associated multimodal MRI frontotemporal network
Source: Nat Commun. 2022 Aug 22;13:4929. doi: 10.1038/s41467-022-32513-8 (PMC9395379; doi:10.1038/s41467-022-32513-8)
Supplement: Supplementary file 1 — Supplementary Information [file 41467_2022_32513_MOESM1_ESM.pdf]

Derivation and utility of schizophrenia polygenic risk associated multimodal MRI frontotemporal network

Shile Qi[1]*, Jing Sui[2]*, Godfrey Pearlson[3], Juan Bustillo[4], Nora I. Perrone-Bizzozero[4], Peter Kochunov[5], Jessica A. Turner[6], Zening Fu[7], Wei Shao[1], Rongtao Jiang[8], Xiao Yang[9], Jingyu Liu[7], Yuhui Du[10], Jiayu Chen[7]*, Daoqiang Zhang[1]*, Vince D. Calhoun[7]

[1]College of Computer Science and Technology, Nanjing University of Aeronautics and Astronautics, Nanjing, China
[2]State Key Laboratory of Cognitive Neuroscience and Learning, Beijing Normal University, Beijing, China
[3]Department of Psychiatry, Yale School of Medicine, New Haven, CT, USA
[4]Departments of Psychiatry and Behavioral Sciences, University of New Mexico, Albuquerque, NM, USA
[5]Department of Psychiatry, University of Maryland School of Medicine, Baltimore, MD, USA
[6]Department of Psychology, Georgia State University, Atlanta, GA, USA
[7]Tri-institutional Center for Translational Research in Neuroimaging and Data Science (TReNDS), [Georgia State University, Georgia Institute of Technology, Emory University], Atlanta, GA, USA
[8]Department of Radiology and Biomedical Imaging, Yale University, New Haven, CT, USA
[9]Huaxi Brain Research Center, West China Hospital of Sichuan University, Chengdu, China
[10]School of Computer & Information Technology, Shanxi University, Taiyuan, China

Supplementary Information

Normal distribution of all the calculated SZ PRS

Supplementary Fig. 1 shows the distribution of polygenic risks scores (PRS) thresholded at P_{SNP}<5.0e-08, P_{SNP}<1.0e-04, P_{SNP}<0.05, and pruned at r^2 < 0.1 and r^2 < 0.2, from both PGC SZ 2 (108 loci) and PGC SZ 3 (270 loci), respectively. We can see that all the PRS were normally distributed within the current UKB sample (N=37,347) for all the conditions: r^2 < 0.1 and r^2 < 0.2, 108 loci and 270 loci, P_{SNP}<5.0e-08, P_{SNP}<1.0e-04 and P_{SNP}<0.05.

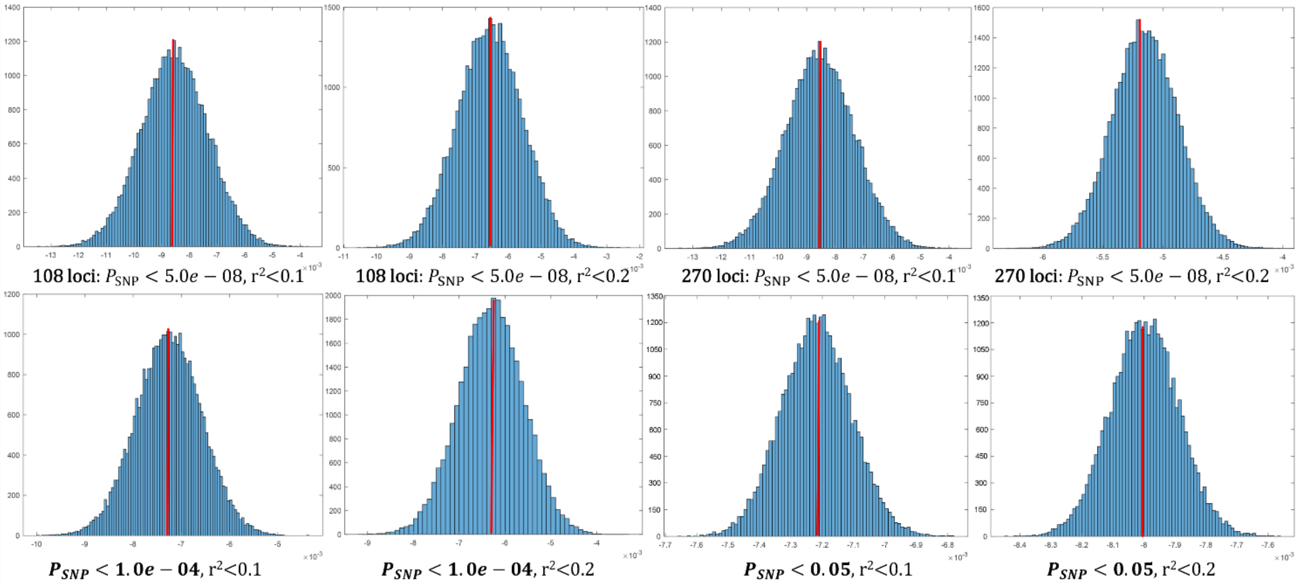

Supplementary Figure 1. Frequency histograms of schizophrenia polygenic risk scores in the UK Biobank at different pruning thresholds (r^2 < 0.1 and r^2 < 0.2) with different P_{SNP} (5.0e-08, 1.0e-04 and 0.05), for both PGC SZ 2 (108 loci) and PGC SZ 3 (270 loci).

Power analysis

We also calculated the statistical power of SZ-PRS and the two MRI features for fusion input (fALFF and GMV) using G*Power software[1] (http://www.softpedia.com/get/Science-CAD/G-Power.shtml). As in this study, the sample size is N=22773 HCs. The effect size of PRS correlates with fALFF loadings is r = 0.074. Given the significance level $\alpha = 0.05$, sample size (N=22773), and the

effect size = 0.074, the statistical power of the correlation is 1. The same method was used to calculate the MRI features, achieving the statistical power of 1 for fALFF and 1 for GMV respectively, which are all high enough to assure accurate and robust conclusions about the correlations detected.

t tests – Correlation: Point biserial model

**Analysis:**	Post hoc: Compute achieved power		
**Input:**	Tail(s)	=	One
	Effect size |ρ|	=	0.074
	α err prob	=	0.05
	Total sample size	=	22773
**Output:**	Noncentrality parameter δ	=	11.1978387
	Critical t	=	1.6449205
	Df	=	22771
	Power (1−β err prob)	=	1.0000000

Supplementary Figure 2. Statistical power generated from the G*Power software.

Weakly association between PRS and the identified components

The correlation between the identified IC and PRS is not very high due to the large sample size. However, compared to the existing studies based on UKB, we found that it is normal that the varaince explained was <1% by correlating SZ-PRS with imaging phenotypes[2,3] under differnet P_{SNP} thresholds and for all the cortical and subcortical areas. This is consistent with a recently published study in Nature 2022 that smaller (sample size) brain wide association studies have reported larger correlations than the largest effects measured in larger samples[4].

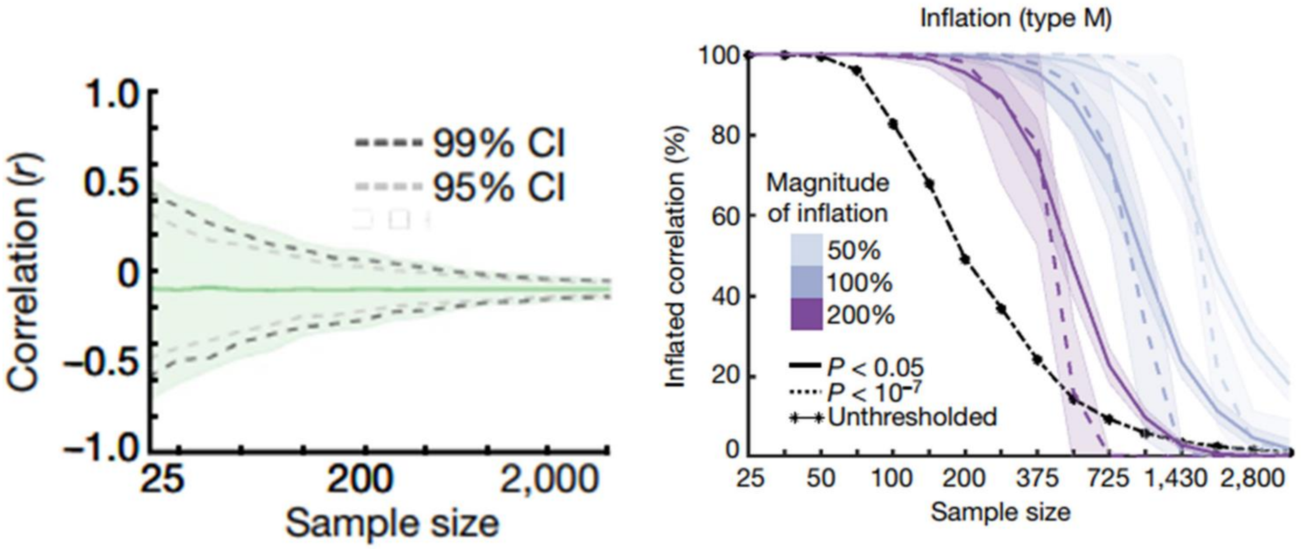

Supplementary Figure 3. As reported in [4], the correlation between cortical thickness and clinical measures decrease as sample size increase (left). And the associations were inflated with small sample size (right).

Existing PRS-imaging association studies based on UKB

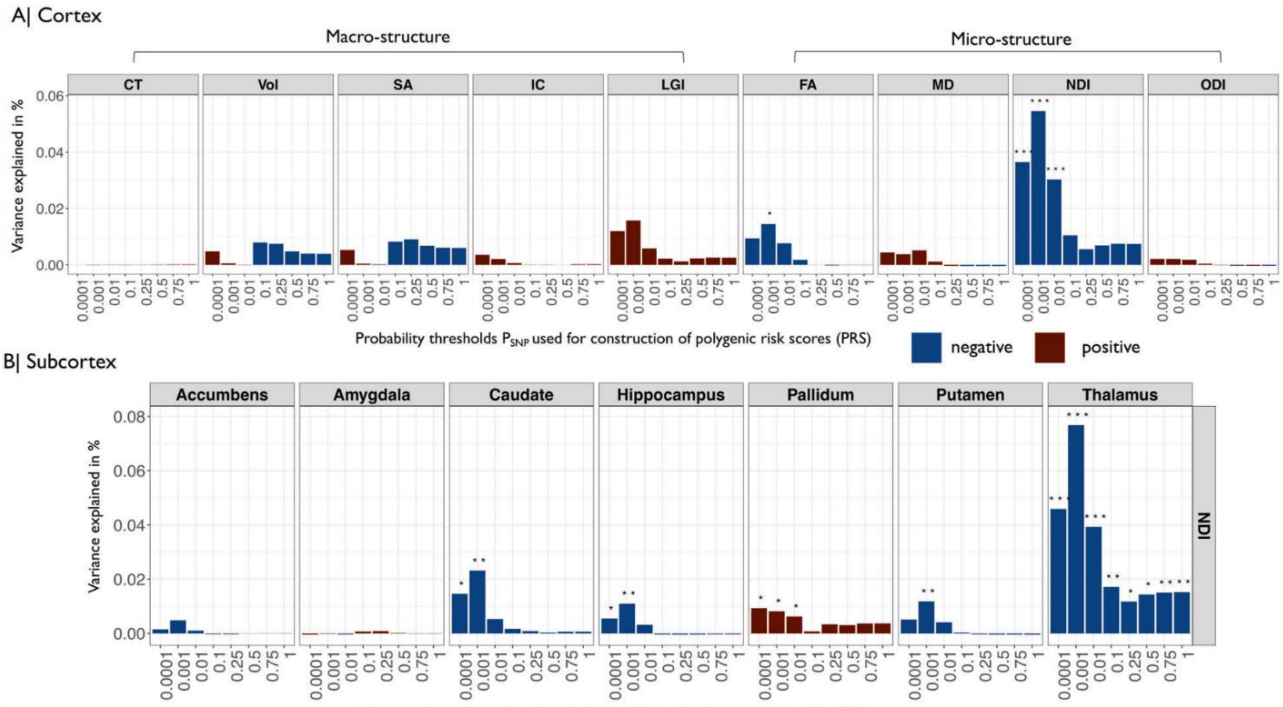

Supplementary Figure[3] 4. Associations between schizophrenia PRS and global cortical and regional subcortical metrics of human brain structure. **A.** Barcharts of variance explained by schizophrenia PRS (R^2, y-axis) constructed at each of eight probability thresholds ($0.0001 \leq P_{\mathrm{SNP}} \leq 1$, x-axis) for each of nine global mean cortical metrics: CT cortical thickness, Vol grey matter volume, SA surface area, IC intrinsic curvature, LGI local gyrification index, FA fractional anisotropy, MD mean diffusivity, NDI neurite density index, ODI orientation dispersion index. **B.** Barcharts of variance explained by PRS (R^2, y-axis) constructed at each of eight probability thresholds ($0.0001 \leq P_{\mathrm{SNP}} \leq 1$, x-axis) for NDI measured at each of seven subcortical regions.

The above PRS and ROI-based single modality association investigations found that the varaince explained was <1% by correlating SZ-PRS with imaging phenotypes[2,3] under differnet P_{SNP} thresholds and for all the cortical and subcortical areas.

For the current study

We have calculated the direct correlation between SZ-PRS and voxel wise MRI features throughout the brain (60758 and 90638 voxels for fALFF and GMV). The maximum absolute correlation r is only 0.03 and 0.028, and the mean r is 0.008 and 0.0006 for fALFF and GMV

respectively.

Apart from the voxel-wise correlation between SZ-PRS and MRI features, we also tested the correlation between the mean values extracted from ALL atlas and SZ-PRS for both fALFF and GMV under different P_{SNP} thresholds. Results (**Supplementary Fig. 5 and Supplementary Table 1**) showed that the variance explained was <1% for all the brain areas under 3 different P_{SNP} thresholds.

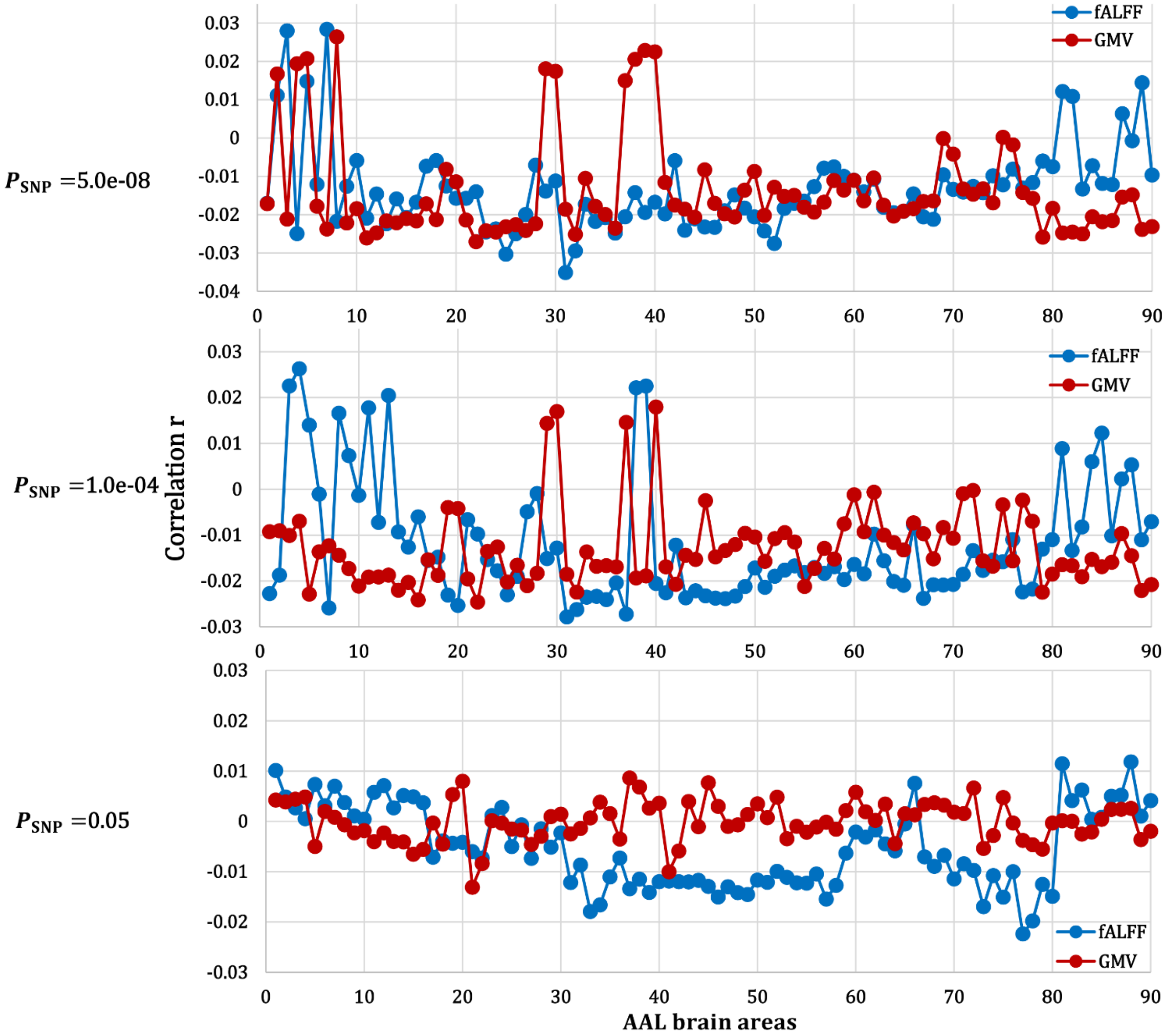

Supplementary Figure 5. Correlations between SZ PRS and the mean values extracted from AAL atlas for both fALFF and GMV under different P_{SNP} thresholds (5.0e-08, 1.0e-04 and 0.05).

Supplementary Table 1. Correlations between PRS and mean values extracted from AAL atlas (90 areas) for both fALFF and GMV under different P_{SNP} thresholds (5.0e-08, 1.0e-04 and 0.05).

Correlation r	P_{SNP} =5.0e-08		P_{SNP} =1.0e-04		P_{SNP} =0.05	
	fALFF	GMV	fALFF	GMV	fALFF	GMV
AAL 1	-0.017	-0.017	-0.022	-0.0092	0.010	0.0042
AAL 2	0.011	0.016	-0.018	-0.0090	0.0048	0.0038
AAL 3	0.027	-0.021	0.022	-0.010	0.0027	0.0044
AAL 4	-0.024	0.019	0.026	-0.006	0.0005	0.0048
AAL 5	0.014	0.020	0.013	-0.022	0.0073	-0.0049
AAL 6	-0.012	-0.017	-0.0010	-0.013	0.0031	0.0019
AAL 7	0.028	-0.023	-0.025	-0.012	0.0070	0.00081
AAL 8	-0.021	0.026	0.016	-0.014	0.0037	-0.00068
AAL 9	-0.012	-0.022	0.0073	-0.017	0.0011	-0.0022
AAL 10	-0.0058	-0.018	-0.0013	-0.021	0.00046	-0.0018
AAL 11	-0.020	-0.026	0.017	-0.019	0.0057	-0.0040
AAL 12	-0.014	-0.024	-0.0072	-0.019	0.0071	-0.0022
AAL 13	-0.022	-0.021	0.020	-0.018	0.0027	-0.0039
AAL 14	-0.015	-0.022	-0.0092	-0.022	0.0051	-0.0040
AAL 15	-0.020	-0.021	-0.012	-0.020	0.0049	-0.0065
AAL 16	-0.016	-0.021	-0.006	-0.024	0.0037	-0.00556
AAL 17	-0.0073	-0.017	-0.015	-0.015	-0.0071	-0.00031
AAL 18	-0.0058	-0.021	-0.014	-0.018	-0.0038	-0.0045
AAL 19	-0.012	-0.0081	-0.023	-0.0039	-0.0043	0.0053
AAL 20	-0.015	-0.011	-0.025	-0.0041	-0.0041	0.0079
AAL 21	-0.015	-0.021	-0.0066	-0.019	-0.0059	-0.013
AAL 22	-0.014	-0.027	-0.0097	-0.024	-0.0072	-0.0083
AAL 23	-0.024	-0.024	-0.015	-0.013	0.0011	0.00014
AAL 24	-0.023	-0.024	-0.017	-0.012	0.0027	-0.00033
AAL 25	-0.030	-0.023	-0.023	-0.020	-0.005	-0.0015
AAL 26	-0.024	-0.022	-0.018	-0.016	-0.00068	-0.0017
AAL 27	-0.019	-0.024	-0.0049	-0.021	-0.0073	-0.0045
AAL 28	-0.0070	-0.022	-0.00091	-0.018	-0.0014	-0.0029

AAL 29	-0.013	0.018	-0.015	0.014	-0.0051	0.00096
AAL 30	-0.011	0.017	-0.012	0.016	-0.0022	0.0014
AAL 31	-0.035	-0.018	-0.027	-0.018	-0.012	-0.0024
AAL 32	-0.029	-0.025	-0.026	-0.022	-0.008	-0.0013
AAL 33	-0.017	-0.010	-0.023	-0.013	-0.017	0.00072
AAL 34	-0.021	-0.017	-0.023	-0.016	-0.016	0.0038
AAL 35	-0.020	-0.019	-0.024	-0.016	-0.011	0.0015
AAL 36	-0.024	-0.023	-0.020	-0.016	-0.0072	-0.0035
AAL 37	-0.020	0.014	-0.027	0.014	-0.013	0.0086
AAL 38	-0.014	0.020	0.022	-0.019	-0.011	0.0068
AAL 39	-0.019	0.022	0.022	-0.018	-0.014	0.0026
AAL 40	-0.016	0.022	-0.020	0.017	-0.011	0.0036
AAL 41	-0.019	-0.011	-0.022	-0.016	-0.011	-0.010
AAL 42	-0.0058	-0.017	-0.012	-0.020	-0.011	-0.0058
AAL 43	-0.024	-0.018	-0.023	-0.014	-0.012	0.0039
AAL 44	-0.021	-0.020	-0.022	-0.015	-0.011	-0.001
AAL 45	-0.023	-0.0083	-0.023	-0.0024	-0.012	0.0076
AAL 46	-0.023	-0.017	-0.023	-0.014	-0.015	0.003
AAL 47	-0.018	-0.019	-0.023	-0.013	-0.012	-0.00098
AAL 48	-0.014	-0.020	-0.023	-0.012	-0.014	-0.00068
AAL 49	-0.018	-0.013	-0.021	-0.009	-0.014	0.0013
AAL 50	-0.020	-0.0087	-0.017	-0.010	-0.011	0.0035
AAL 51	-0.024	-0.020	-0.021	-0.015	-0.012	0.00075
AAL 52	-0.027	-0.012	-0.018	-0.010	-0.0099	0.0048
AAL 53	-0.018	-0.015	-0.017	-0.0094	-0.011	-0.0034
AAL 54	-0.017	-0.014	-0.016	-0.011	-0.012	-0.00096
AAL 55	-0.016	-0.018	-0.018	-0.021	-0.012	-0.0021
AAL 56	-0.012	-0.019	-0.017	-0.017	-0.010	-0.0011
AAL 57	-0.0078	-0.016	-0.018	-0.012	-0.015	-0.00015
AAL 58	-0.0075	-0.011	-0.016	-0.015	-0.012	-0.0015
AAL 59	-0.01	-0.013	-0.019	-0.007	-0.0063	0.0021
AAL 60	-0.010	-0.011	-0.016	-0.0011	-0.0020	0.0058
AAL 61	-0.014	-0.016	-0.018	-0.009	-0.0031	0.0019

AAL 62	-0.010	-0.010	-0.0097	-0.00065	-0.0017	0.0002
AAL 63	-0.018	-0.017	-0.015	-0.0099	-0.0044	0.0034
AAL 64	-0.019	-0.020	-0.020	-0.011	-0.0058	-0.0044
AAL 65	-0.019	-0.019	-0.020	-0.013	-0.00051	0.0015
AAL 66	-0.014	-0.018	-0.0078	-0.0073	0.0075	0.0013
AAL 67	-0.020	-0.016	-0.023	-0.0096	-0.0070	0.0033
AAL 68	-0.021	-0.016	-0.020	-0.015	-0.0089	0.0037
AAL 69	-0.0095	-9.6e-05	-0.020	-0.0083	-0.0067	0.0032
AAL 70	-0.013	-0.0041	-0.020	-0.010	-0.011	0.0018
AAL 71	-0.014	-0.013	-0.018	-0.00096	-0.0084	0.0015
AAL 72	-0.012	-0.014	-0.013	-0.00024	-0.0097	0.00665
AAL 73	-0.014	-0.013	-0.017	-0.015	-0.016	-0.0053
AAL 74	-0.0098	-0.016	-0.015	-0.016	-0.010	-0.0027
AAL 74	-0.012	0.00021	-0.015	-0.0033	-0.015	0.0047
AAL 76	-0.0080	-0.0017	-0.010	-0.015	-0.0099	-0.00031
AAL 77	-0.013	-0.014	-0.022	-0.0023	-0.022	-0.0037
AAL 78	-0.011	-0.015	-0.021	-0.0069	-0.019	-0.0046
AAL 79	-0.0059	-0.025	-0.013	-0.022	-0.012	-0.0055
AAL 80	-0.0074	-0.018	-0.010	-0.018	-0.014	-0.00032
AAL 81	0.012	-0.024	0.0088	-0.016	0.011	0.00015
AAL 82	0.010	-0.024	-0.013	-0.016	0.0041	4.9e-05
AAL 83	-0.013	-0.025	-0.0082	-0.019	0.0062	-0.0025
AAL 84	-0.0071	-0.020	0.0060	-0.015	0.00042	-0.0021
AAL 85	-0.011	-0.021	0.012	-0.016	0.00081	0.00042
AAL 86	-0.012	-0.021	-0.010	-0.015	0.0050	0.0023
AAL 87	0.0063	-0.015	0.0022	-0.0096	0.0052	0.0024
AAL 88	-0.00068	-0.014	0.0053	-0.014	0.011	0.0025
AAL 89	0.014	-0.023	-0.011	-0.022	0.0010	-0.003
AAL 90	-0.0096	-0.023	-0.0070	-0.020	0.0041	-0.0019

Supplementary Table 2. Anatomical information of the identified PRS-associated joint components.

fMRI_IC1 Area	Brodmann Area	volume (cc)	random effects: Max Value (x, y, z) R/L
**Positive**			
Middle/Inferior Frontal Gyrus	6, 8, 9, 10, 44, 45, 46, 47	6.6/0.0	3.0 (-36, 56, 8)/NaN
Superior/Middle Temporal Gyrus	21, 22, 38, 39	6.3/0.4	2.9 (-62, -29, 1)/2.1 (65, -35, 2)
**Negative**			
Posterior Cingulate	23, 30, 31	3.2/4.0	3.0 (-9, -66, 14)/3.1 (12, -60, 14)
Middle Occipital Gyrus	18, 19	2.4/2.7	2.7 (-21, -87, 15)/2.6 (21, -90, 16)
Thalamus		0.1/0.3	2.1 (-6, -14, 15)/2.5 (9, -11, 14)
Lingual Gyrus	17	1.4/0.9	2.3 (-24, -88, -6)/2.8 (18, -90, -1)
**sMRI_IC1 Area**	**Brodmann Area**	**volume (cc)**	**random effects: Max Value (x, y, z)**
**Positive**			
Hippocampal Gyrus	19, 30	0.6/0.2	5.2 (-24, -38, 5)/2.6 (24, -41, 5)
Insula	13	2.2/1.3	4.9 (-30, 15, 10)/5.1 (33, 18, 10)
**Negative**			
Superior/Middle/Inferior Temporal Gyrus	13, 19, 20, 21, 22, 37, 39, 41	4.9/2.9	8.8 (-48, -26, -4)/4.6 (48, -26, -4)
Fusiform Gyrus	20, 37	1.0/1.0	4.3 (-45, -41, -11)/5.6 (45, -41, -11)
Parahippocampal Gyrus	30, 36	2.3/1.5	3.7 (-24, -38, -6)/3.3 (27, -35, -6)
Insula	13, 47	0.3/0.5	3.3 (-45, 12, -1)/3.6 (42, -26, -1)

Linear projection

In this study, we further tested the replicability of the associations detected between PRS and multimodal components within UKB, *i.e.*, whether the association between PRS and the same pattern can be detected in independent SZ dataset, by performing cross-site linear projection analysis. When combing the four independent SZ cohorts: BSNIP, fBIRN, COHRE and PK, there are totally N=290 diagnosed SZ patients with PRS, fMRI, sMRI available for replication. Starting with fMRI, after fusion with PRS within UKB, $\boldsymbol{S}_{\text{fMRI}}^{\text{UKB}} = \left(\boldsymbol{A}_{\text{fMRI}}^{\text{UKB}}\right)^{-}\boldsymbol{X}_{\text{fMRI}}^{\text{UKB}}$ was generated in the discovery cohort (UKB). For

the validation cohort (SZ), we obtained the mixing matrix by linear projection as: $\boldsymbol{A}_{\text{fMRI}}^{\text{SZ}} = \boldsymbol{X}_{\text{fMRI}}^{\text{SZ}} \times \left(\boldsymbol{S}_{\text{fMRI}}^{\text{UKB}}\right)^{-}$ ($\boldsymbol{S}_{\text{fMRI}}^{\text{UKB}}$ from UKB was used as spatial maps for SZ). The same approach was used for sMRI projection from UKB to SZ. Pearson correlation analysis was used to calculate the association between PRS and the loadings of the target components (the same index in UKB, *i.e.*, IC1) in SZ. Results showed that the association between PRS and frontotemporal pattern identified in UKB can still be examined in independent SZ patients, which means that the association can be replicated.

Null pattern

In order to see the null pattern, we permuted the reference vector (PRS) in the supervised fusion analysis. The goal is to compute the null model of spatial patterns that are observed by chance. To do this we hold imaging variables (e.g. $[\boldsymbol{X}_1, \ \boldsymbol{X}_2]$) constant, and permute the PRS against them. Thus each $\boldsymbol{X}_{\text{i}}$ is randomly paired with a reference. This permuted reference was then used as reference in the supervised fusion analysis (MCCAR+jICA). By repeating this process, a large number of times (500), we obtain 500 fMRI-sMRI covarying patterns associated with the permuted reference. We also record the number of times each voxel occurs. Here we presented the most frequently occurring voxels (those which occur more than 70% of the time) associated with the permuted PRS, as shown in **Supplementary Fig. 6b**. Note that the permuted null model of spatial pattern is different from the comprised frontotemporal system (no hippocampus complex and insular detected in null pattern), confirming that the identified PRS pattern is specific to the PRS but not a random null pattern.

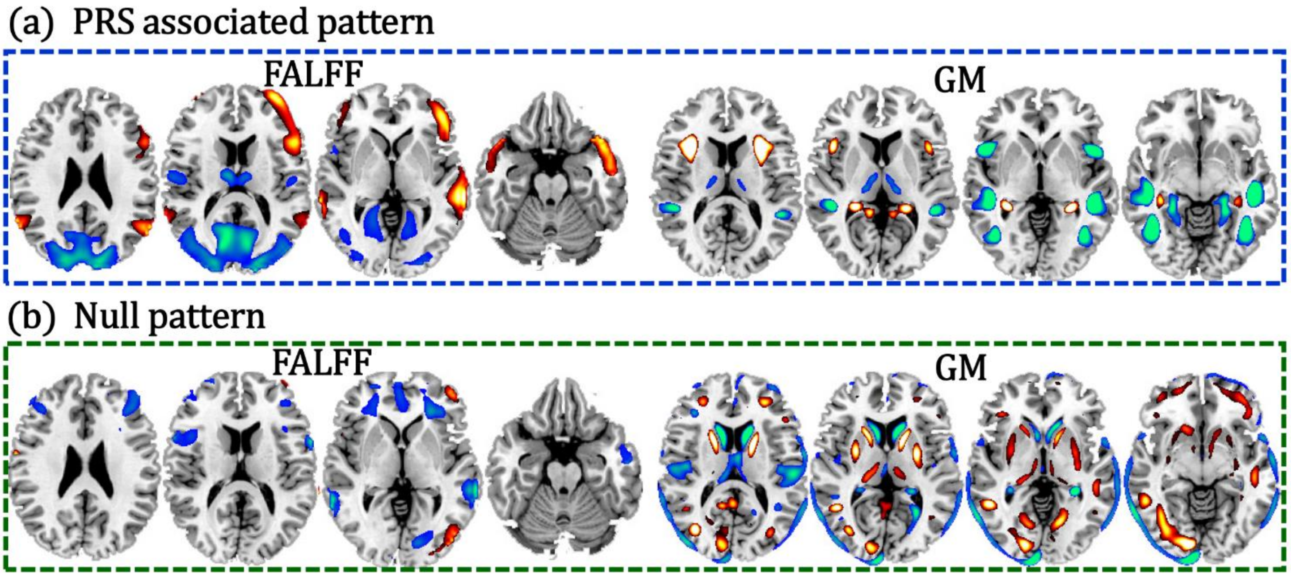

Supplementary Figure 6. (**a**) The fALFF+GMV covarying pattern associated with PRS. (**b**) The most frequently occurring (voxels with more than 60% occurrences) covarying pattern associated with 500 times permuted PRS.

Spatial similarity

Here, take fALFF components as an example. We calculated the spatial correlation of the identified PRS-associated components between **Fig. 3a** and **Fig. 3b** with only voxels masked at |Z|>***T*** (threshold). First, the spatial maps were transformed into Z scores and masked at |Z|>2. Then we obtained two masks from **Fig. 3a** (mask_a) and **Fig. 3b** (mask_b) respectively, which were used to perform the voxel selection. Only voxels that fell in the union of the masks (mask_a ∪ mask_b, regardless of positive and negative) were used to calculate the spatial correlation. Thus total number of voxels in calculating the spatial correlation is greatly reduced, *e.g.*, from n= 153594 (the whole brain voxels) to m=5635 (***T***=2). Spatial correlation was finally performed on these commonly identified voxels (m=5635) between **Fig. 3a** and **Fig. 3b**.

Supplementary Table 3. Spatial similarity between Fig. 3a and Fig. 3b-f.

Correlation (*r* value)	Fig. 3b	Fig. 3c	Fig. 3d	Fig. 3e	Fig. 3f
Fig. 3a fALFF	0.91	0.88	0.87	0.82	0.82
Fig. 3a GMV	0.92	0.89	0.88	0.83	0.82

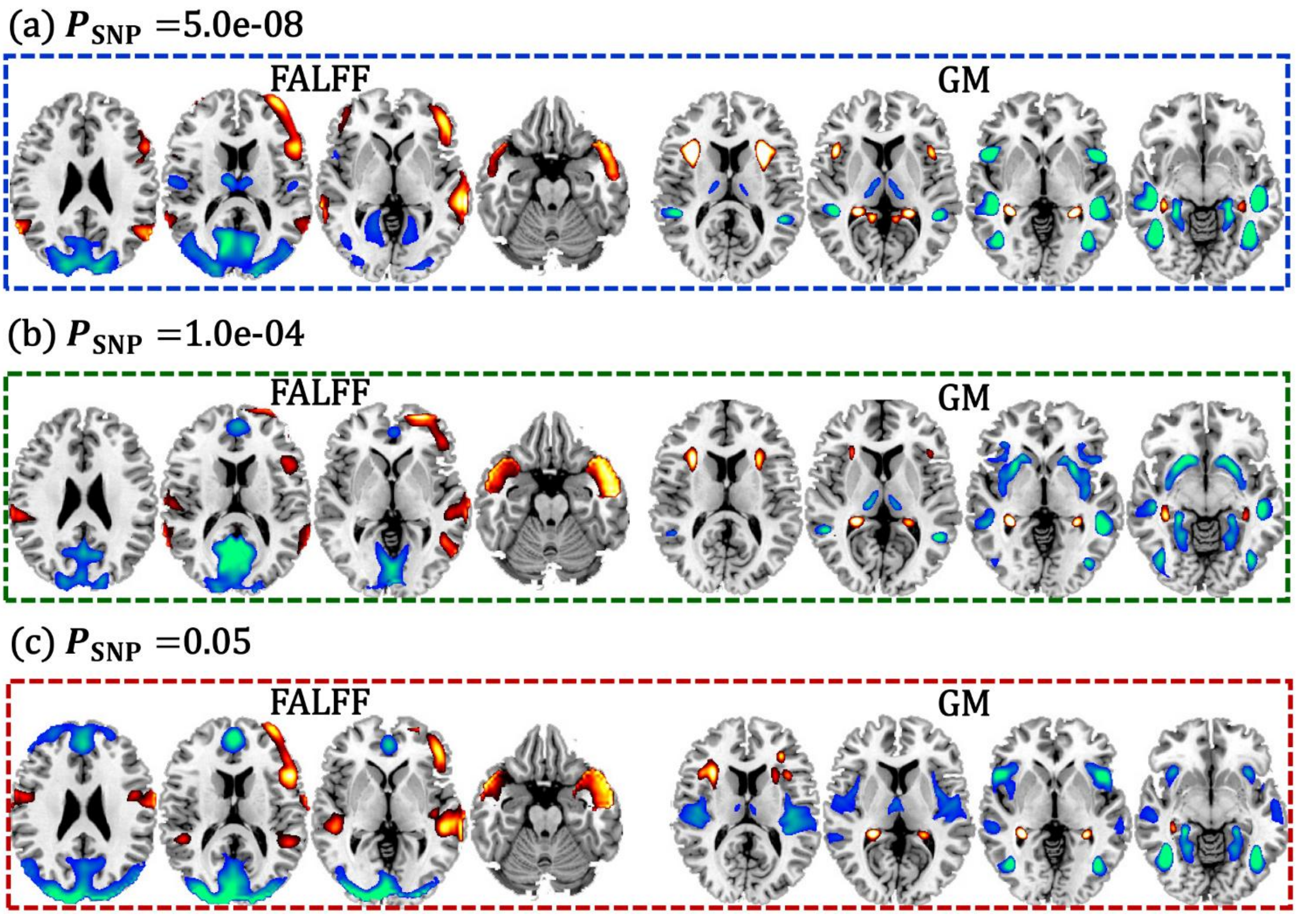

Supplementary Figure 7. Fusion with PRS under different P_{SNP} thresholds. (a) P_{SNP}<5.0e-08; (b) P_{SNP}<1.0e-04; (c) P_{SNP}<0.05. The positive fALFF in MIFC, SMTC, negative fALFF in PCC and MOC, accompanied with positive GMV in anterior insula and hippocampus, and negative GMV in MITC, and para-hippocampus were all well replicated under different P_{SNP} thresholds. The spatial similarity among these PRS-associated patterns are displayed in the following table.

Supplementary Table 4. Spatial similarity between Supplementary Fig. 7a and Supplementary Fig. 7b-c.

**Correlation (*r* value)**	**Supplementary Fig. 7b**	**Supplementary Fig. 7c**
Supplementary Fig. 7a fALFF	0.53	0.64
Supplementary Fig. 7a GMV	0.72	0.58

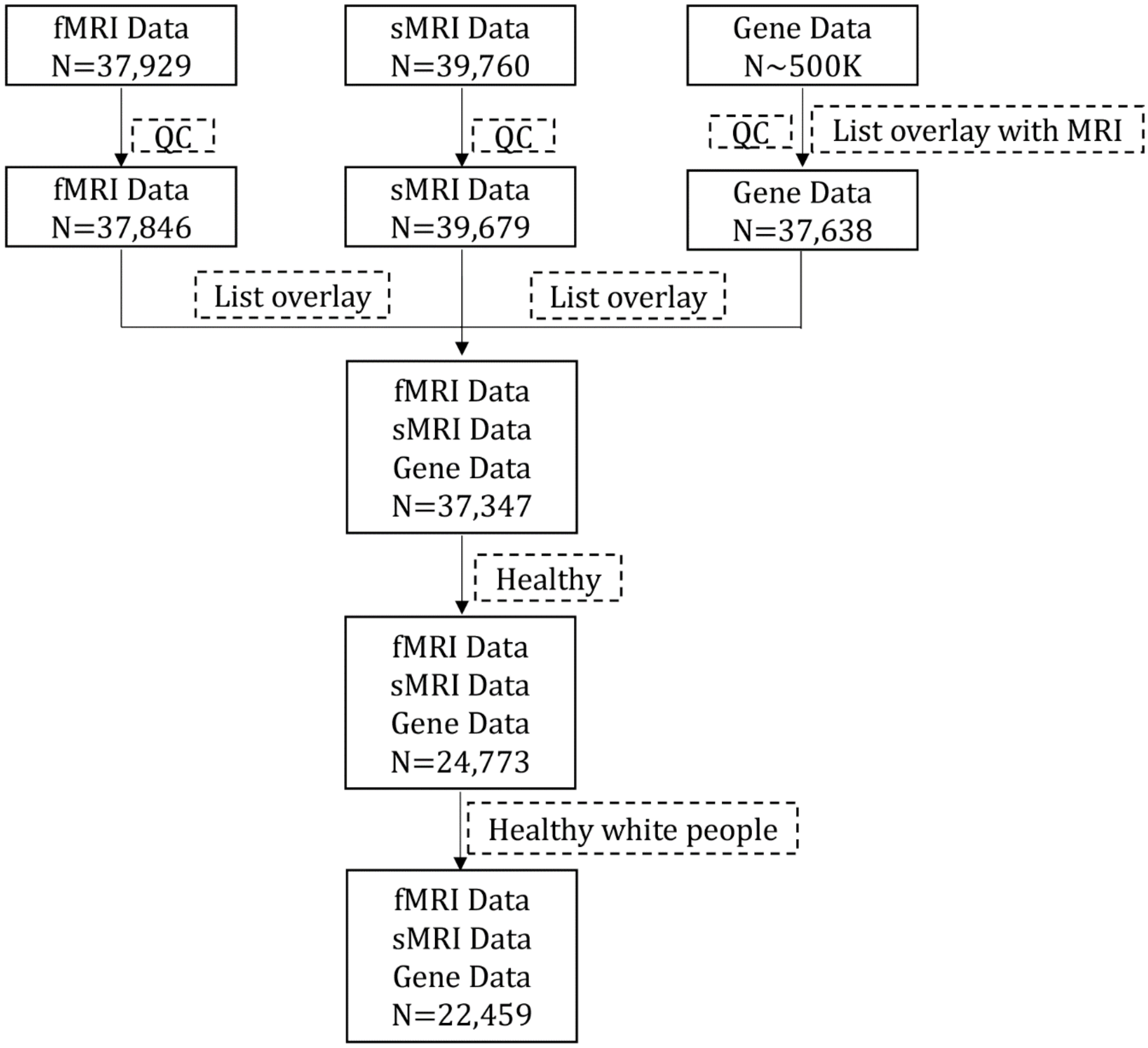

Supplementary Figure 8. Sample selection details. QC: quality control.

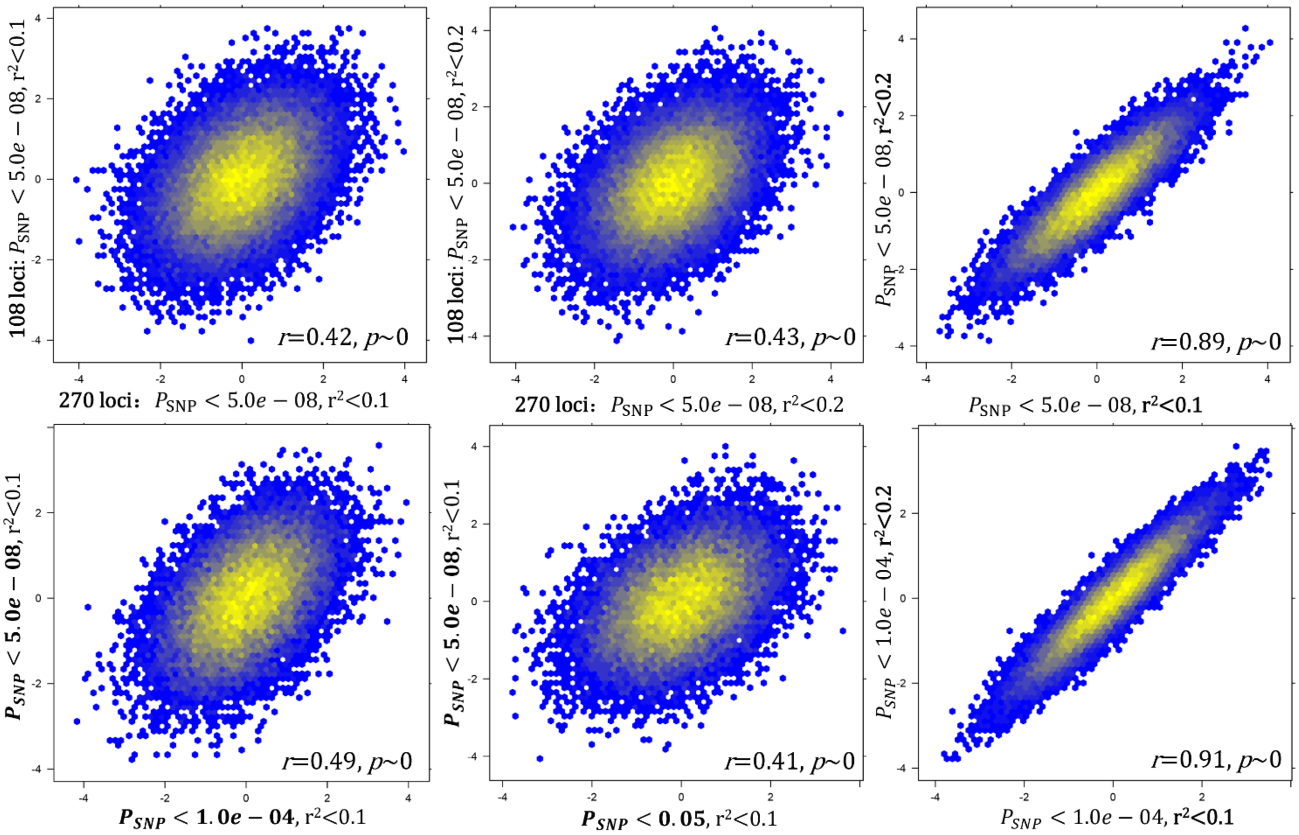

Supplementary Figure 9. 2D density plot of PRS between PGC SZ 2 (108 loci) and PGC SZ 3 (270 loci), and between different P_{SNP} and pruning thresholds. It shows that the PRS calculated from PGC SZ 2 (108 loci) and PGC SZ 3 (270 loci) are highly correlated (r~0.4), as well as between different P_{SNP} thresholds (r~0.4), and even more higher correlation between different pruning thresholds (r~0.9). So, different pruning thresholds generate more highly correlated SZ PRS than different P_{SNP}. Pearson correlation was used to calculated the correlation between PRS under different thresholds in Supplementary Fig. 7.

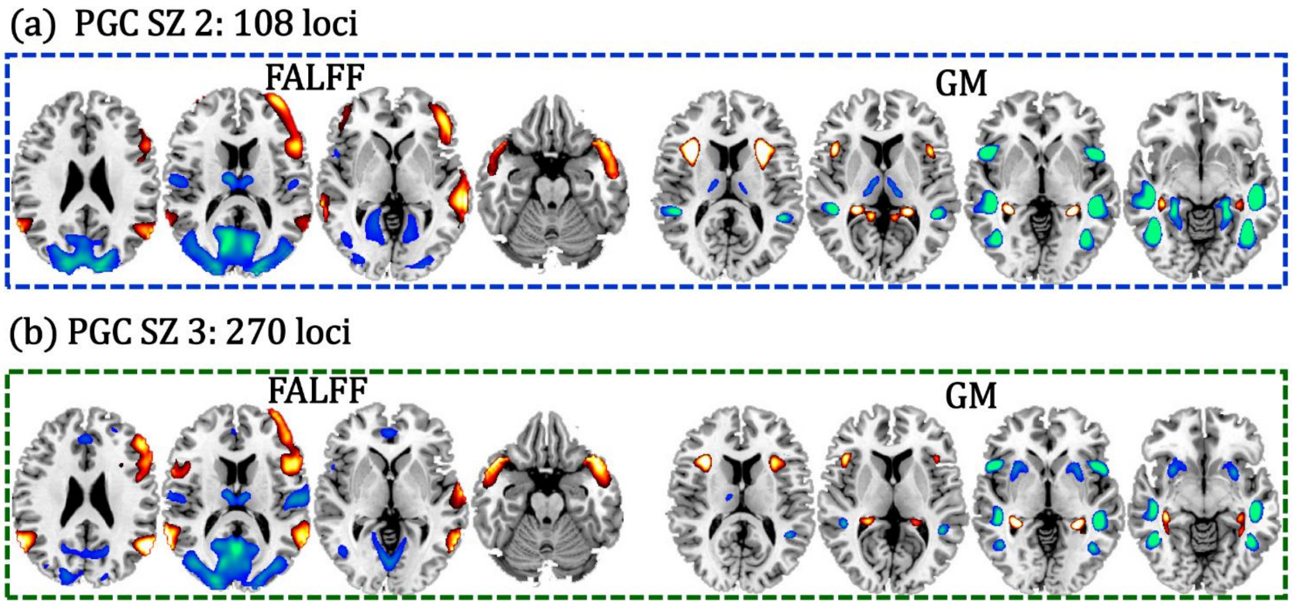

Supplementary Figure 10. (**a**) The fALFF+GMV covarying pattern associated with PRS calculated from PGC SZ 2: 108 loci. (**b**) The fALFF+GMV covarying pattern associated with PRS calculated from PGC SZ 3: 270 loci.

The PRS-associated pattern was replicated with positive fALFF in middle and inferior frontal cortex, superior and middle temporal cortex, negative fALFF in thalamus, posterior cingulate cortex, and middle occipital cortex, accompanied with positive GMV in anterior insula and hippocampus, and negative GMV in middle insula, superior/middle/inferior temporal cortex, and fusiform gyrus. The spatial similarities between PGC SZ 2 and PGC SZ 3 of the PRS-pattern are r=0.89 and r=0.85 for fALFF and GMV components, respectively.

Site effect

Site effect on PRS-MRI fusion within UKB

For the MRI imaging data, there are three sites available in UKB, including Cheadle, Reading and Newcastle. We performed the PRS-guided fusion for each site separately to test the similarity of the identified PRS-associated frontotemporal pattern. Dice index, equation (1) was used to calculate the overlap percentage of the spatial maps between sites. Dice index is a statistical validation for comparing the spatial similarity of binary images, for example in image segmentation accuracy assessment. We calculated the Dice index of the identified PRS-associated component between two cohorts using only voxels masked at |Z|>2, resulting in two masks from UKB (mask_UKB) and Cheadle/Reading/Newcastle (mask_Cheadle/Reading/Newcastle) respectively. Only voxels that fell into the union of the masks (mask_UKB ∪ mask_Cheadle) were used to calculate the cross-cohort similarity as shown in equation (1).

$$\text{Dice index} = 2\frac{V(A \cap B)}{V(A)+V(B)} \quad (1)$$

Results (**Supplementary Fig. 11 and Supplementary Table 5**) showed that there were high spatial similarities among Cheadle, Reading, Newcastle and UKB. The Dice index for GM and fALFF components were >0.70, suggesting that there was high overlap percentage of the spatial maps cross different sites within UKB. Thus we do not believe that site would be a major confounding factor with respect to the identified PRS frontotemporal multimodal pattern.

Supplementary Table 5. Spatial similarity between UKB and Cheadle/Reading/Newcastle sites.

Dice	Cheadle	Reading	Newcastle
UKB fALFF	0.92	0.75	0.77
UKB GMV	0.91	0.70	0.81

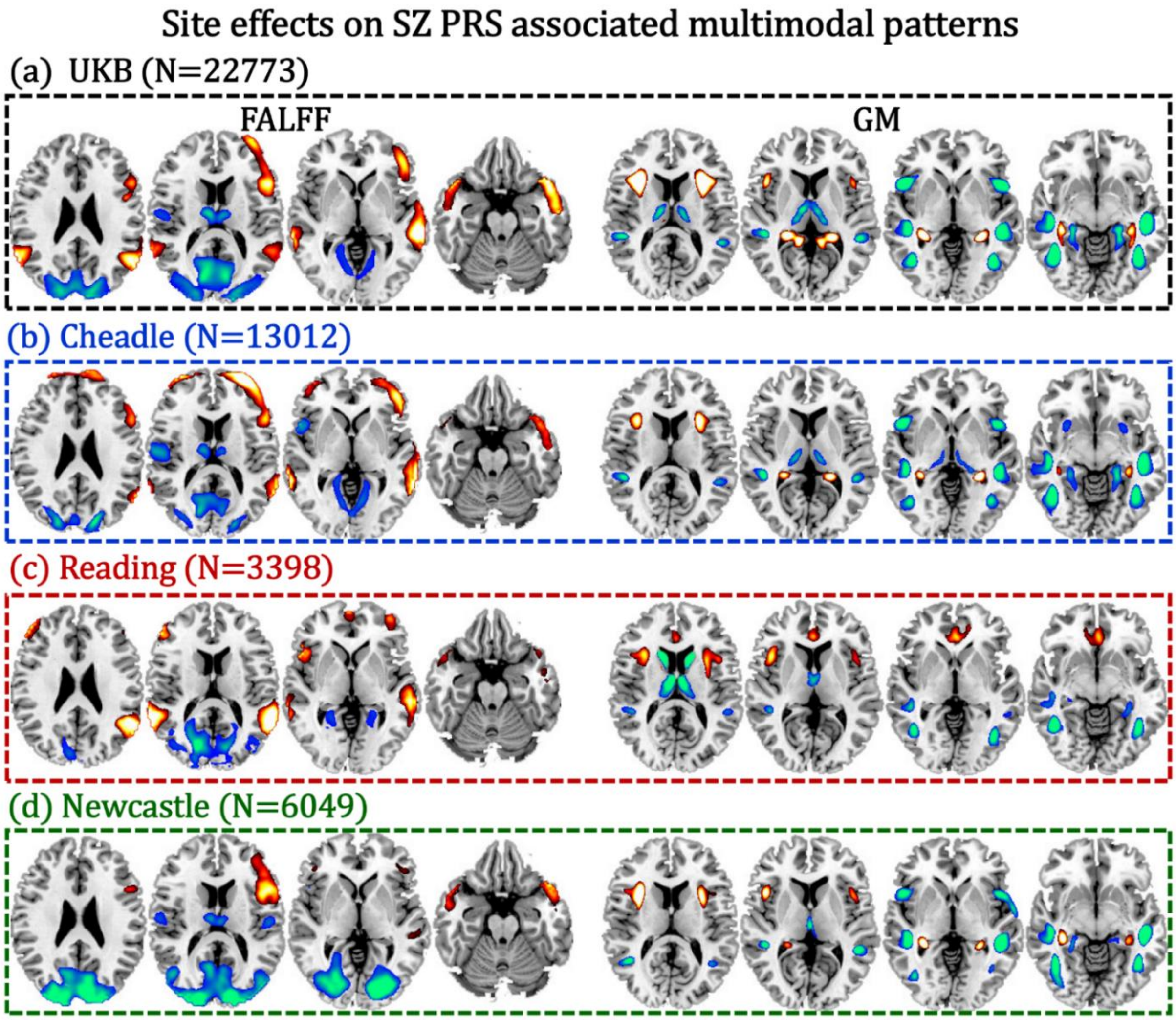

Supplementary Figure 11. PRS pattern for UKB (**a**), Cheadle (**b**), Reading (**c**), and Newcastle (**d**). The spatial similarities were > 0.70 across UKB, Cheadle, Reading, and Newcastle for both fALFF and GMV components.

Supplementary Table 6. Site differences on cognition and PANSS scores for fBRIN and BSNIP cohorts.

Anova (p value)	Cognition	PANSS positive	PANSS negative
BSNIP	0.13	0.093	0.165
fBIRN	0.08	0.93	0.86

Motion effect

Motion on preprocessing

To control confounding effects of motion artifact, several strategies were conducted. In the preprocessing procedure for fMRI, we despiked the fMRI data: nuisance covariates (6 head motions + cerebrospinal fluid [CSF] + white matter [WM]) + global signal were regressed out via a general linear model from the voxel time series. The outlier subjects with framewise displacements (FD) exceeding 1.0 mm, as well as head motion exceeding 2.5 mm of maximal translation (in any direction of x, y or z) or 1.0° of maximal rotation were excluded. Results indicate all FDs (mean framewise displacements, mean of root of mean square frame-to-frame head motions assuming 50 mm head radius [5]) for all subjects were 1 mm at every time point. We performed correlation analysis between clinical scores with mean FD, as displayed in **Supplementary Table 7**. There is no significant correlations between the mean FD and age, gender, PRS, handiness and ethnicity.

Correlations between head motion and other clinical measures

Supplementary Table 7. Correlation between head motion and other clinical measures in UKB, as well as the loadings for fMRI and sMRI.

Correlation (*p* value)	Age	Sex	PRS0.1	PRS0.2	Handiness	Ethnicity	fMRI_IC	sMRI_IC
Mean FD	*p*=0.9	*p*=0.21	*p*=0.09	*p*=0.32	*p*=0.53	*p*=0.62	*p*=0.82	*p*=0.54

PRS pattern on UKB subset with head motion <0.2mm

We also exclude subjects with >0.2mm FD to get a subset of UKB (N = 13490, 60% subjects' head motion <0.2mm) to perform the fusion with PRS to test whether the identified multimodal frontotemporal pattern can be replicated. Result (**Supplementary Fig. 12b**) show that the identified PRS-associated pattern (frontotemporal cortex and thalamus in fALFF, accompanied with thalamus, hippocampus, para-hippocampus and temporal cortex in GMV) can be validated on UKB subset with

FD<0.2mm. This means that the head motion is not a major confounding factor for our current fusion results.

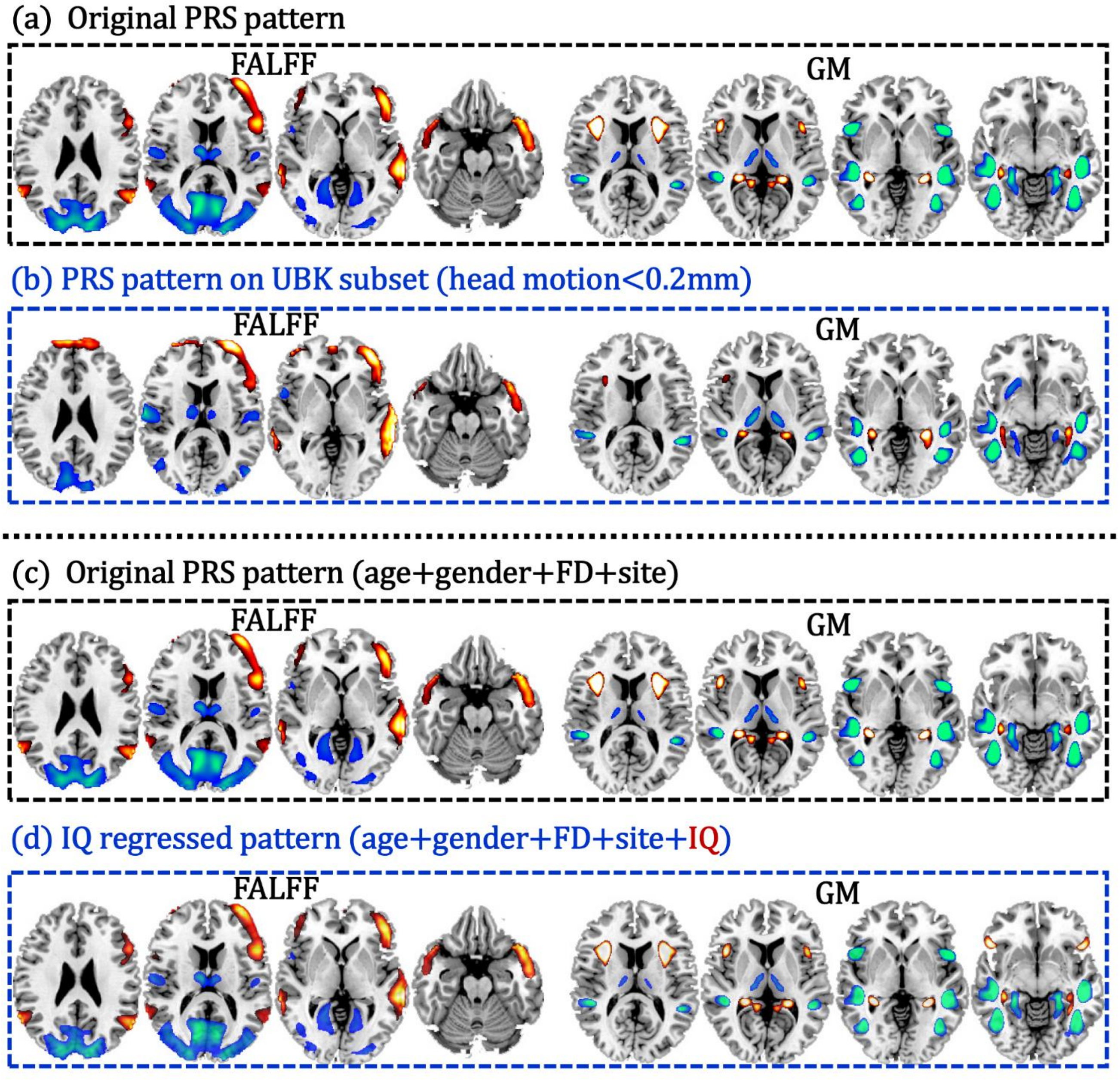

Supplementary Figure 12. (**a, c**) The original PRS-associated fALFF+GMV covarying pattern for UKB. (**b**) The PRS pattern on UKB subset with head motion <0.2mm. (**d**) The PRS pattern after regressing out IQ.

Group differences of mean FD between SZ and HC

We have calculated the group differences of mean FD between HC and SZ across the 4 SZ cohorts

included in this study. Note that there is no significant differences between patients and controls on mean FD for all the 4 SZ cohorts, namely,

BSNIP, HC: mean=0.22±0.11mm, SZ: 0.26±0.24mm, two sample t-test: $p = 0.25$

FBIRN, HC: mean=0.25±0.18mm, SZ: 0.27±0.21mm, two sample t-test: $p = 0.65$

COBRE, HC: mean=0.22±0.12mm, SZ: 0.21±0.11 mm, two sample t-test: $p = 0.77$

MPRC, HC: mean=0.12±0.22mm, SZ: 0.18±0.10mm, two sample t-test: $p = 0.15$

Partial correlation

Partial correlation has been proposed as an alternative approach for removing spurious shared variance in correlation analysis [6]. Here, we also performed partial correlation analysis between the identified component and PRS by regressing out mean FD. Result show that the significant level is not changed by mean FD (p = 5.2e-30* for fALFF, p = 2.3e-28* for GMV as in **Fig. 2b**).

fALFF not functional connectivity

Furthermore, fMRI data were spatially smoothed with a 6 mm full width half max (FWHM) Gaussian filter. To calculate fractional amplitude of low frequency fluctuations (fALFF)[7], the sum of the amplitude values in the 0.01 to 0.08Hz low-frequency power range was divided by the sum of the amplitudes over the entire detectable power spectrum (range: 0–0.25Hz)[8]. So, the fusion analysis was conducted on the spatial maps of fALFF not the function connectivity. Previous fMRI studies found that head motion was sensitive to functional connectivity analysis[9-13]. However, the current fusion analysis was conducted on the spatial maps of fALFF not functional connectivity. While it is the functional connectivity derived from rs-fMRI that is more sensitive to head motion[9-13].

Collectively, considering there was no group difference in head motion between SZ and HC, and no significant correlation between mean FD and PRS, and the partial correlation between the identified component and PRS still significant after regressing out mean FD, and the PRS-pattern was replicated on UKB subset with head motion <0.2mm, the current fusion analysis was based on fALFF not

functional connectivity, we believe that micro-motion was not a major factor affecting the current results.

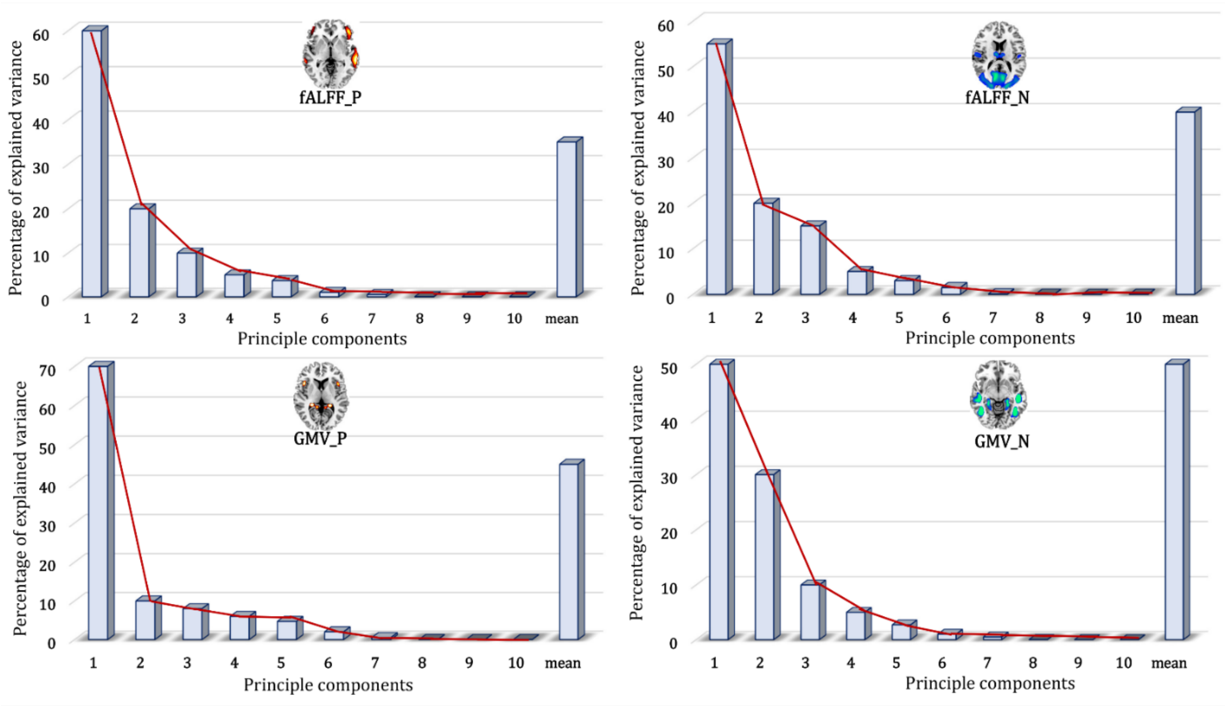

Supplementary Figure 13. Percentage of explained variance comparing PCA components and the mean extracted from the identified PRS-associated ROIs.

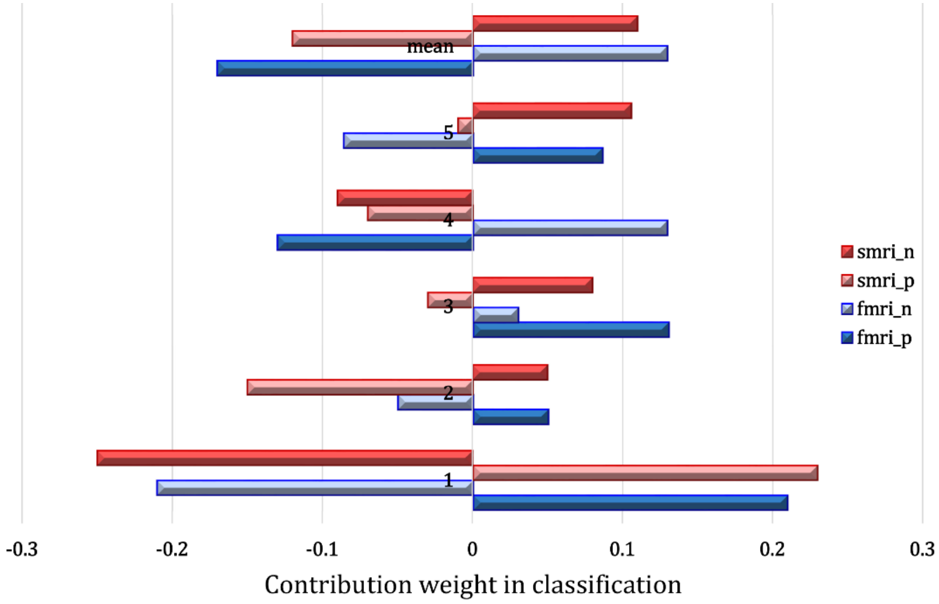

Supplementary Figure 14. Beta weights for classification comparing PCA components and the mean extracted from the identified PRS-associated ROIs.

Supplementary Table 8. Correlation between the mean the 5 PCs.

Correlation	1st	2nd	3rd	4th	5th
r	0.53	0.02	0.08	0.27	-0.04
p	1.2e-20	0.80	0.25	1.7e-04	0.58

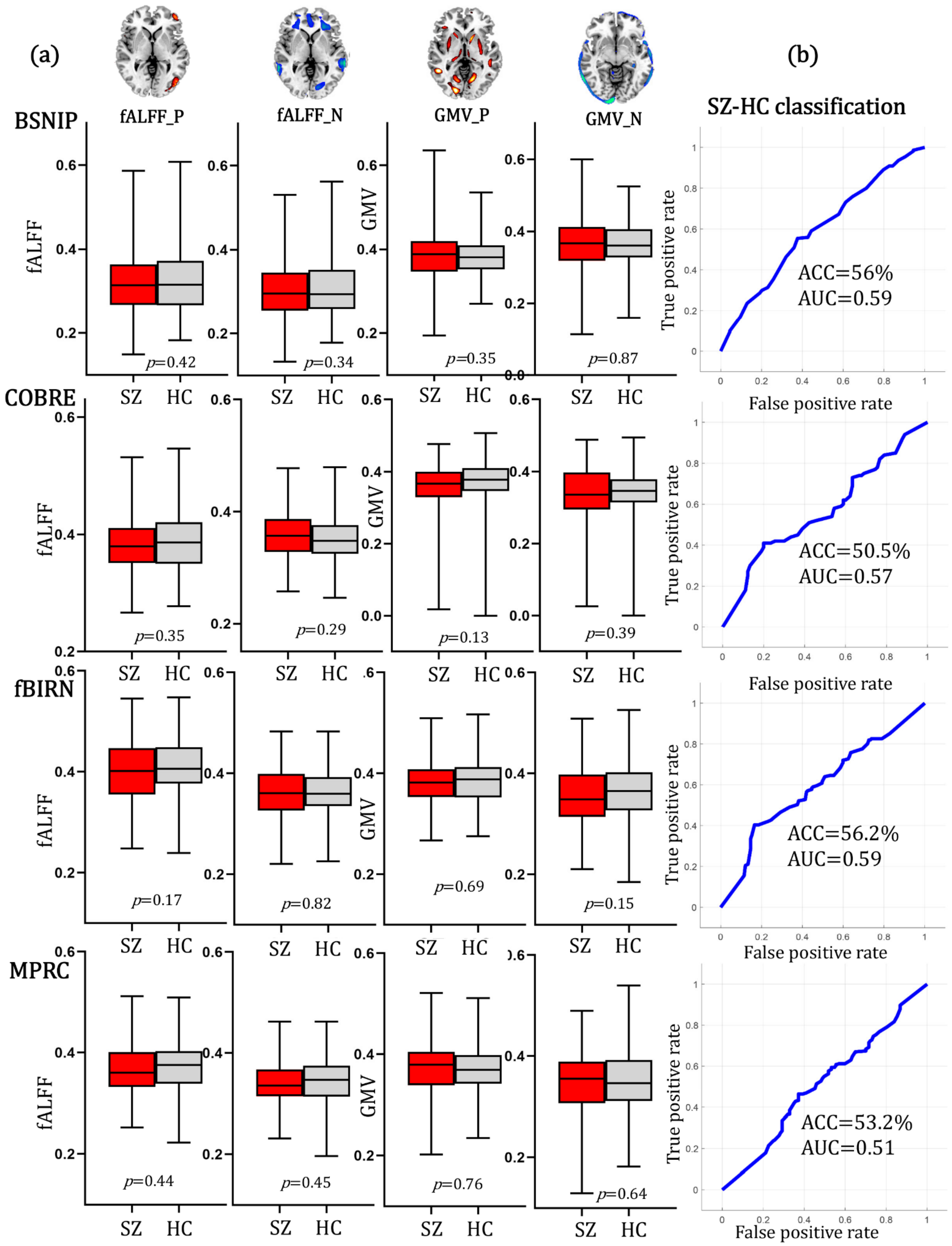

Supplementary Figure 15. (a) Group differences between SZ and HC of the permuted pattern in independent BSNIP-1 (SZ: N=178; HC: N=220), COBRE (SZ: N=100; HC: N=90), fBIRN (SZ: N=164; HC: N=157) and MPRC (SZ: N=164; HC: N=157) cohorts, respectively. The minima, maxima

and the mean were displayed in the box plots. (b) ROC curves of the classification results between SZ and HC for BSNIP-1, COBRE, fBIRN and MPRC cohorts, respectively.

Classification on scanning site

There are 4 independent SZ cohorts (BSNIP, COBRE, fBIRN, MPRC) included in our current study. However, different SZ cohorts consist different number of sites. There are 5 sites for BSNIP, 1 site for COBRE, 7 sites for fBIRN and 3 sites for MPRC. Since COBRE is a single site, so the classification on scanning sites are performed for BSNIP (class=3), fBIRN (class=7) and MPRC (class=3). The mean fALFF/GMV plus the first 5PCs within positive and negative PRS-associated brain networks were used as feature input and sites was treated as labels in the SVM classifications. Results (**Supplementary Fig. 16**) showed that all the classification accuracies were approximated as around 50% as a random distributed accuracy (the more number of site the lower classification accuracy). This means that site is not a major confounding factor for the current SZ-HC classification result.

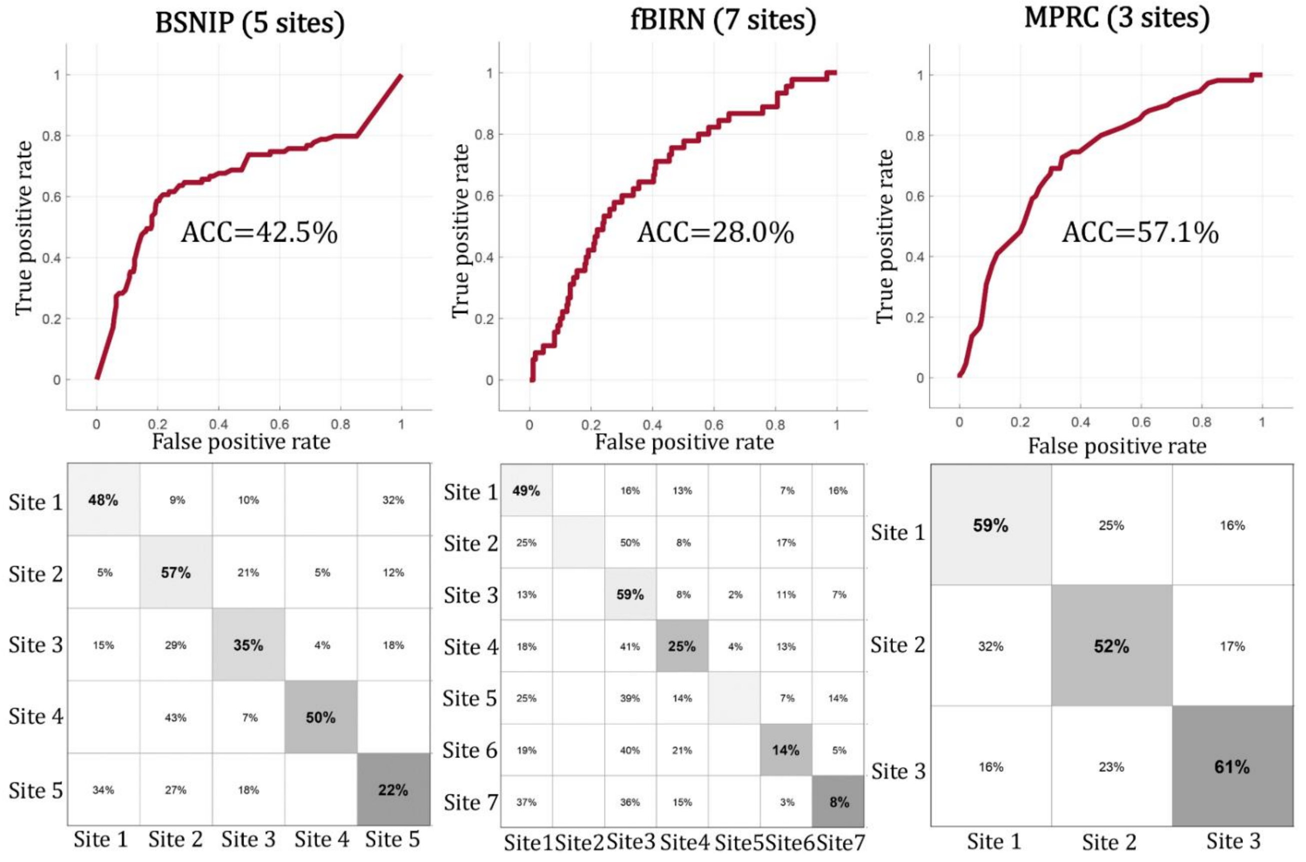

Supplementary Figure 16. The classification results on scan sites for BSNIP, fBIRN and MPRC cohorts. Upper row represents ROC; lower row represents confusion matrix.

Site effect on PRS, PANSS and cognition

Anova test (site was used as covariate) showed that there was no site difference of PRS (p=0.96) for UKB data. The site differences of PANSS and cognition for independent SZ cohorts were shown in the following Table. Since clinical scores are not available for MPRC cohort, and COBRE is a single site cohort, so these two cohorts were not included in the following Table.

Collectively, all above results indicate that site is not a major confounding factor for PRS pattern, and classification.

Multimodal imaging parameters and preprocessing

Resting state fMRI

UKB: The UKB datasets were collected in three sites (Cheadle, Newcastle and Reading) using a standard EPI sequence, including Siemens 3-Tesla Siemens scanner (TR/TE = 735/39 ms, voxel spacing size = 2.4 ×2.4 ×2.4 mm, FOV = 88 ×88 ×64 matrix). GE-EPI with ×8 multi-slice acceleration, no iPAT, flip angle 52°, fat saturation.

BSNIP:

Supplementary Table 9. Scanning information for BSNIP samples.

Site	TR ms	TE ms	Flip angle	Slices number	Voxel Size (mm)	Time points
Baltimore	2210	30	70°	36	3.4×3.4×4	140
Chicago	1775	27	60°	29	3.4×3.4×5	210
Dallas	1500	27	60°	29	3.4×3.4×5	210
Boston	1720	23	60°	29	3.8×3.8×5	210
Hartford	1500	27	70°	29	3.4×3.4×5	210

FBIRN: The fBIRN dataset was collected from seven sites. The same rsfMRI parameters were used across all sites: a standard gradient EPI sequence, repetition time (TR)/echo time (TE) = 2,000/30

ms, voxel spacing size = 3.4375 × 3.4375 × 4 mm, slice gap = 1 mm, flip angle (FA) = 77°, field of view (FOV) = 220 × 220 mm, and a total of 162 volume. Six of the seven sites used 3-Tesla Siemens Tim Trio scanners, and one site used a 3-Tesla General Electric Discovery MR750 scanner.

MPRS: The MPRC dataset was collected in three sites using a standard EPI sequence, including Siemens 3-Tesla Siemens Allegra scanner (TR/TE = 2,000/27 ms, voxel spacing size = 3.44 × 3.44 × 4 mm, FOV = 220 × 220 mm, and 150 volumes); 3-Tesla Siemens Trio scanner (TR/TE = 2,210/30 ms, voxel spacing size = 3.44 × 3.44 × 4 mm, FOV = 220 × 220 mm, and 140 volumes); and 3-Tesla Siemens Tim Trio scanner (TR/TE = 2,000/30 ms, voxel spacing size = 1.72 × 1.72 × 4 mm, FOV = 220 × 220 mm, and 444 volumes).

COBRE: The COBRE dataset was collected in one site using a standard EPI sequence with TR/TE = 2,000/29 ms, voxel spacing size = 3.75 × 3.75 × 4.5 mm, slice gap = 1.05 mm, FA = 75°, FOV = 240 × 240 mm, and a total of 149 volumes. Data were collected using a 3-Tesla Siemens Tim Trio scanner.

ABIDE II: Resting-state fMRI and sMRI were available from each participant who met inclusion criteria. Detailed image acquisition parameters can be found in the ABIDE website https://.fcon_1000.projects.nitrc.org/indi/abide/abide_II.html for ABIDE II.

MDD: The resting state fMRI data were collected on a 3T whole MR scanner (Achieva, Philips, Netherlands) using an eight-channel phased-array head coil. During scanning, foam padding and earplugs were used to minimize the head movement and scanner noise. All participants underwent a high resolution three-dimensional T1-weighted, sagittal, magnetization-prepared rapid gradient echo (MPRAGE) sequence with the following parameters: repetition time (TR) = 8.4 ms; echo time (TE) = 3.9 ms; flip angle = 7°; in-plane matrix resolution = 256×256; field of view (FOV) = 256×256 mm; voxel size = 1×1×1 mm^3; thickness = 1 mm; number of slices = 188.

For fMRI, standard preprocessing based on statistical parametric mapping (SPM12, http://www.fil.ion.ucl.ac.uk/spm/) under MATLAB 2019 environment included the following: 1) the first five dummy scans have been discarded to retain only scans when the scanner approach to the

steady state 2) slice timing correction; 3) realignment;4) normalization to the EPI template with 3 × 3 × 3mm^3 resolution; 5) spatial smoothing using a 6-mm full width half-maximum Gaussian kernel; After that, nuisance covariates (6 head motions + cerebrospinal fluid [CSF] + white matter [WM]) + global signal were regressed out via a general linear model from the voxel time series, and 7) calculation of fractional amplitude of low frequency fluctuations (fALFF). The same fMRI preprocessing pipeline was applied for UKB, BSNIP, fBIRN, COBRE and MPRC cohorts.

Structure MRI

UKB: High-resolution anatomical MR images were acquired, including a three-dimensional T1-weighted magnetization prepared gradient echo sequence (MPRAGE) based on the Alzheimer's disease Neuroimaging Initiative protocol. The resolution of structural image is 1 × 1 × 1 mm (TR = 735 msec, TE = 39 msec, FOV: 208 × 256 × 256 matrix) and required 5 minutes for acquisition.

FBIRN: High-resolution structural brain scans were also acquired on six 3T Siemens Tim Trio System and one 3T GE Discovery MR750 scanner using standardized sequences. Siemens MP-RAGE scan parameters were TR/TE/TI = 2300/2.94/1100 ms, flip angle = 9°, resolution = 256 × 256 × 160. GE IR_SPGR scan parameters were TR/TE/TI = 5.95/1.99/45 ms, FA= 12°, resolution = 256 × 256 × 166. All scans covered the entire brain with FOV = 220 mm^2, voxel size = 0.86 × 0.86 × 1.2 mm^3, sagittal scan plane, GRAPPA/ASSET acceleration factor = 2, and NEX = 1.

COBRE: High-resolution anatomical MR images were acquired, including a three-dimensional T1-weighted MPRAGE protocol. The resolution of structural image is 1 × 1 × 1 mm (TR/TE = 2,000/29 ms, FOV = 240 × 240 mm).

MDD: A total of 240 volumes of echo planar images were obtained axially with a gradient echo EPI sequence with the following parameters: TR = 2000 ms; TE = 30 ms; in-plane matrix resolution = 64×64; field of view = 240×240 mm; number of slices = 38. For the resting scan, subjects were instructed to lie still with eyes closed.

For the sMRI data was normalized to MNI space using the unified segmentation method in

SPM12, resliced to 3 × 3 × 3 mm, and segmented into gray matter (GM), white matter (WM), and cerebral spinal fluid (CSF) using modulated normalization algorithms, resulting outputs as gray matter volume (GMV). Then the GMV were smoothed using a Gaussian kernel with a full width at half maximum (FWHM) = 6 mm. Subject outlier detection was further performed using a spatial Pearson correlation with the template image to ensure that all subjects were properly segmented.

Cognitive measures

Brief Assessment of Cognition in Schizophrenia (BACS). This battery assesses multiple cognitive functions, with a global neuropsychological functioning composite score integrating over multiple domains provided the best measure of psychosis-related cognitive deviation. Age and sex-stratified normative data were used to compute these composite scores for each participant.

Computerized Multiphasic Interactive Neuro-cognitive System (CMINDS)[14]. Neurocognitive domain z-scores were calculated from computerized neuropsychological tests. The CMINDS includes computerized neuropsychological tasks that are structurally and functionally similar to standard paper-and-pencil neuropsychological tasks and allows for immediate electronic raw data capture and automated scoring of test results. The CMINDS-based[14] cognitive domains, based on comparable tests to those assessed by the MCCB, were as follows: (1) *Speed of Processing*. This domain score was based on the mean of (a) the log-transformed, negated (worse performance is lower) elapsed time (in seconds) during *Trails A*, (b) the number of correct in set responses in 60 seconds on trial 1 of the *Category Fluency Test –Animals*, and (c) the number of correct responses during the *Symbol Digit Association Test* z-scores; (2) *Attention/Vigilance*. This domain score was based on the *d*-prime across blocks A–C of the *Continuous Performance Test z* -scores; (3) *Working Memory.* This domain score was based on the mean of (a) the sum of the number of correct on the *Visual Spatial Sequencing Test* – Forward and Backward condition, and (b) the total correct on the *Letter Number Span z* -scores; (4) *Verbal Learning.* This domain score was based on the total number of correctly recalled target words for all three trials on the *Semantic Verbal Learning Test* z-scores; (5) *Visual Learning.* This domain

score was based on the square-transformed total of the *Visual Figure Learning Test z*-scores, and (6) *Reasoning/Problem Solving*. This domain score was based on the square transformed *Maze Solving Test* total score *z*-scores. Finally, the CMINDS composite score was defined as the mean of all six normalized domain scores."

MATRICS Consensus Cognitive Battery (MCCB) system was also launched by NIMH, and contains one more domain (social cognition) than CMINDS. As reported earlier[14], CMINDS is very similar to MATRICS on measuring cognitive deficits in SZ. The differences in details between CMINDS and MCCB tasks have been previously cited[14].

Supplementary Table 10. Demographics for BSNIP subjects.

**Measures**		**SZ**	**HC**	**p**
**Number**		178	220	
**Age**		34.534±12.0	38.836±12.6	5.60e-04
**Gender**		54F/127M	129M/90F	5.97e-09
**BACS**	Composite	-1.719±1.4	0.005±1.1	1.83e-32
	Verbal memory	-1.045±1.3	0.043±1.1	3.56e-14
	Digit sequencing	-1.214±1.1	-0.073±1.1	3.04e-20
	Token motor	-1.355±1.2	0.040±1.1	1.29e-26
	Verbal fluency	-0.685±1.1	0.143±1.0	1.80e-12
	Symbol coding	-1.365±1.1	-0.043±1.0	1.26e-28
	Tower	-0.819±1.4	-0.008±1.1	2.54e-09
**PANSS**	Negative	16.256±6.1	7.600±0.9	2.22e-12
	Positive	16.345±5.7	8.200±1.6	2.24e-05

Supplementary Table 11. Demographics for FBIRN subjects.

Measures		SZ	HC	*p*
**Number**		164	157	
**Age**		39.006±11.3	37.516±11.3	0.242
**Gender**		41F/120M	45F/112M	0.523
**CMINDS**	Composite	-1.604±1.3	0.010±1.0	2.70e-29
	Speed of processing	-1.305±1.1	0.005±1.0	1.74e-24
	Attention/vigilance	-1.403±1.4	0.001±1.0	2.52e-20
	Working memory	-1.196±1.1	0.010±1.0	1.04e-21
	Verbal learning	-1.313±1.2	0.030±1.0	1.10e-23
	Visual learning	-1.058±1.1	0.011±1.0	4.42e-17
	Reasoning/problem solving	-0.895±1.3	-0.003±1.0	1.87e-11
**PANSS**	Negative	14.360±5.6	NA	NA
	Positive	15.503±5.1	NA	NA

Supplementary Table 12. Demographics for COBRE subjects.

Measures		SZ	HC	p
**Number**		100	90	
**Age**		38.490±14.1	38.000±11.6	0.793
**Gender**		22F/78M	25F/65M	0.361
**MCCB**	Composite	32.707±12.9	49.921±8.7	2.68e-19
	Speed of processing	36.053±11.7	53.807±8.6	2.60e-23
	Attention/vigilance	37.266±12.9	49.987±9.2	2.93e-12
	Working memory	39.221±12.0	48.916±10.7	5.10e-08
	Verbal learning	38.379±8.3	45.530±8.3	4.64e-08
	Visual learning	37.684±12.2	46.024±10.7	2.71e-06
	Reasoning/problem solving	44.495±10.5	55.741±8.1	2.55e-13
**PANSS**	Negative	14.620±5.2	NAN	NAN
	Positive	15.020±4.6	NAN	NAN

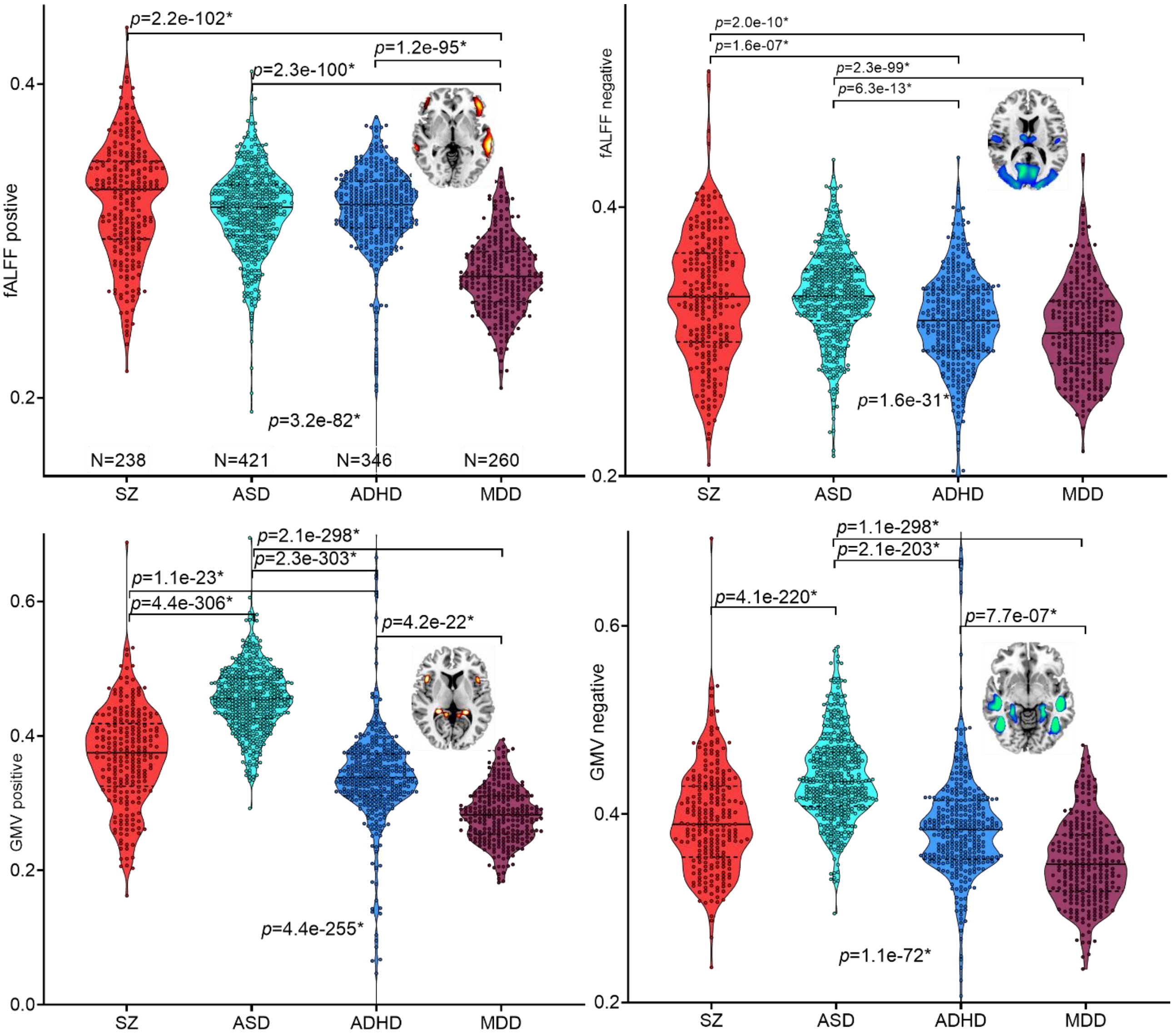

Supplementary Figure 17. Group differences among disorders. Two-tailed two-sample T test was used to calculate the group differences between any two groups of patients in Supplementary Fig. 15. Although there were significant group differences among different disorders, site would be a major concern when interpreting this result.

Feature selection and classification

The identified PRS-associated fALFF+GMV components were separated into positive (Z>0) and negative (Z<0) brain networks based on the Z-scored brain maps. Thus 4 PRS-associated brain features (fALFF_positive, fALFF_negative, GMV_positive, GMV_negative) were obtained by averaging fALFF/GMV in these networks. Apart from the mean values, the first 5 principel component

(the first PC from PCA) from decomposition of fALFF/GMV matrics within the positive/negative networks were also included in the following classification analysis.

Linear SVM implemented in the Classification Learner app (MATLAB2019) was used as the classification model for all the classification analyses. Instead of manually selecting hyper-parameters, we used hyperparameter optimization within the Classification Learner app to automate the selection of hyperparameter values. By default, the Classification Learner app performs hyperparameter tuning by using Bayesian optimization. Note that there are imbalanced subject number in MPRC cohort. We did re-sampling for large sample size groups in order to get comparable sample size for each group. Undersampling by randomly removing some subjects from SZ group was performed to get comparable subject numbers (n=150) with the HC group. The above random re-sampling was repeated 200 times. Within each re-sampling, an unbiased 10-fold cross-validation framework, in which nine of the ten folds were used as the training data and the remaining fold was used as the testing data, was applied. We tested the classification ability of the identified PRS associated multimodal features in differentiating between SZ and HC across 4 independent SZ cohorts.

Supplementary References

1. Faul, F., Erdfelder, E., Lang, A.G. & Buchner, A. G*Power 3: a flexible statistical power analysis program for the social, behavioral, and biomedical sciences. *Behavior research methods* **39**, 175-191 (2007).
2. Neilson, E.*, et al.* Impact of Polygenic Risk for Schizophrenia on Cortical Structure in UK Biobank. *Biological psychiatry* **86**, 536-544 (2019).
3. Stauffer, E.M.*, et al.* Grey and white matter microstructure is associated with polygenic risk for schizophrenia. *Molecular psychiatry* (2021).
4. Marek, S.*, et al.* Reproducible brain-wide association studies require thousands of individuals. *Nature* **603**, 654-660 (2022).
5. Allen, E.A.*, et al.* A baseline for the multivariate comparison of resting-state networks. *Frontiers in systems neuroscience* **5**, 2 (2011).
6. Smith, S.M.*, et al.* Network modelling methods for FMRI. *Neuroimage* **54**, 875-891 (2011).
7. Zou, Q.H.*, et al.* An improved approach to detection of amplitude of low-frequency fluctuation (ALFF) for resting-state fMRI: fractional ALFF. *Journal of neuroscience methods* **172**, 137-141 (2008).

8. Turner, J.A., *et al.* A multi-site resting state fMRI study on the amplitude of low frequency fluctuations in schizophrenia. *Front Neurosci* **7**, 137 (2013).
9. Satterthwaite, T.D., *et al.* An improved framework for confound regression and filtering for control of motion artifact in the preprocessing of resting-state functional connectivity data. *Neuroimage* **64**, 240-256 (2013).
10. Satterthwaite, T.D., *et al.* Impact of in-scanner head motion on multiple measures of functional connectivity: relevance for studies of neurodevelopment in youth. *Neuroimage* **60**, 623-632 (2012).
11. Ciric, R., *et al.* Benchmarking of participant-level confound regression strategies for the control of motion artifact in studies of functional connectivity. *Neuroimage* **154**, 174-187 (2017).
12. Ciric, R., *et al.* Mitigating head motion artifact in functional connectivity MRI. *Nature protocols* **13**, 2801-2826 (2018).
13. Power, J.D., *et al.* Ridding fMRI data of motion-related influences: Removal of signals with distinct spatial and physical bases in multiecho data. *Proceedings of the National Academy of Sciences of the United States of America* **115**, E2105-E2114 (2018).
14. van Erp, T.G., *et al.* Neuropsychological profile in adult schizophrenia measured with the CMINDS. *Psychiatry research* **230**, 826-834 (2015).
