# Supplementary material for: Derivation and utility of schizophrenia polygenic risk associated multimodal MRI frontotemporal network
Source: Nat Commun. 2022 Aug 22;13:4929. doi: 10.1038/s41467-022-32513-8 (PMC9395379; doi:10.1038/s41467-022-32513-8)
Supplement: Supplementary file 3 — Reporting Summary [file 41467_2022_32513_MOESM3_ESM.pdf]

nature portfolio

Corresponding author(s): Shile Qi

Last updated by author(s): Jul 2, 2022

Reporting Summary

Nature Portfolio wishes to improve the reproducibility of the work that we publish. This form provides structure for consistency and transparency in reporting. For further information on Nature Portfolio policies, see our Editorial Policies and the Editorial Policy Checklist.

Statistics

For all statistical analyses, confirm that the following items are present in the figure legend, table legend, main text, or Methods section.

n/a	Confirmed	
☐	☒	The exact sample size (n) for each experimental group/condition, given as a discrete number and unit of measurement
☐	☒	A statement on whether measurements were taken from distinct samples or whether the same sample was measured repeatedly
☐	☒	The statistical test(s) used AND whether they are one- or two-sided<br>*Only common tests should be described solely by name; describe more complex techniques in the Methods section.*
☐	☒	A description of all covariates tested
☐	☒	A description of any assumptions or corrections, such as tests of normality and adjustment for multiple comparisons
☐	☒	A full description of the statistical parameters including central tendency (e.g. means) or other basic estimates (e.g. regression coefficient) AND variation (e.g. standard deviation) or associated estimates of uncertainty (e.g. confidence intervals)
☐	☒	For null hypothesis testing, the test statistic (e.g. F, t, r) with confidence intervals, effect sizes, degrees of freedom and P value noted<br>*Give P values as exact values whenever suitable.*
☒	☐	For Bayesian analysis, information on the choice of priors and Markov chain Monte Carlo settings
☒	☐	For hierarchical and complex designs, identification of the appropriate level for tests and full reporting of outcomes
☐	☒	Estimates of effect sizes (e.g. Cohen's d, Pearson's r), indicating how they were calculated

Our web collection on statistics for biologists contains articles on many of the points above.

Software and code

Policy information about availability of computer code

Data collection	No software was used in data collection.
Data analysis	SPM12 (version 2) was used for fMRI and sMRI preprocessing. MATLAB 2019b was used to do the statistical and classification analysis. GPower 3.1 was used to calculate the power of the statistical significance. GraphPad Prism 8 was used to generate Fig. 6-7. Fusion ICA Toolbox (FIT, https://trendscenter.org/software/fit) was used to perform the supervised fusion analysis.

For manuscripts utilizing custom algorithms or software that are central to the research but not yet described in published literature, software must be made available to editors and reviewers. We strongly encourage code deposition in a community repository (e.g. GitHub). See the Nature Portfolio guidelines for submitting code & software for further information.

Data

Policy information about availability of data

All manuscripts must include a data availability statement. This statement should provide the following information, where applicable:

- Accession codes, unique identifiers, or web links for publicly available datasets
- A description of any restrictions on data availability
- For clinical datasets or third party data, please ensure that the statement adheres to our policy

Data availability:
The UKB, ADHD and ASD multimodal data used in the present study can be accessed upon application from UKB, ADHD-200 and ABIDE consortiums. The FBIRN, BSNIP-1, COBRE, MPRC and MDD data are protected and are not publicly available due to data privacy. The fBIRN, BSNIP-1, COBRE and MPRC data can be accessed upon request to Dr. Vince D. Calhoun. The MDD data can be accessed upon request to Dr. Jing Sui.

Field-specific reporting

Please select the one below that is the best fit for your research. If you are not sure, read the appropriate sections before making your selection.

- [x] Life sciences
- [ ] Behavioural & social sciences
- [ ] Ecological, evolutionary & environmental sciences

For a reference copy of the document with all sections, see nature.com/documents/nr-reporting-summary-flat.pdf

Life sciences study design

All studies must disclose on these points even when the disclosure is negative.

Sample size	Healthy whites in UKB data (N=22,459) was used to identify PRS-associated multimodal pattern. The criterion for subject selection include: healthy, white, and each of them completed the genotype and multimodal MRI data. Subjects passed MRI quality control were selected. No specific sample size calculation was performed.
Data exclusions	Subjects that have any ICD-10 coded neurological or psychiatric diseases, congenital neurological diseases, that reported themselves that they were told to have a specific neurological/psychiatric disease (which may or may not have been ICD-coded), non-white, with incomplete MRI data were excluded.
Replication	Four independent SZ cohorts (fBIRN, COBRE, BSNIP, MPRC) were used to do the replication analysis. The group difference tests, the classification between SZ and HC, the prediction of symptom and cognition were replicated across four independent SZ cohorts.
Randomization	There is only one group (healthy subjects) in UKB. For fBIRN, COBRE, BSNIP and MPRC, there are SZ and control groups. Subjects with mean FD exceeding 1 mm, and head motion exceeding 2.5 mm of maximal translation (in any direction of x, y or z) or 2.5o of maximal rotation throughout the course of scanning were excluded. We further regressed out six head motion parameters (3 translations and 3 rotations) cerebrospinal fluid [CSF] + white matter [WM] + global signal in resting state fMRI preprocessing. The correlation between mean FD and PRS was not significant (p>0.05, Supplementary Table 5). In addition, mean FD, site, gender and age were regressed out from fALFF/GMV feature matrices prior to the primary fusion analysis.
Blinding	Subjects from fBIRN, COBRE, BSNIP and MPRC were grouped based on whether they diagnosed as SZ or not. The investigators were blinded to group allocation during data collection and/or analysis.

Reporting for specific materials, systems and methods

We require information from authors about some types of materials, experimental systems and methods used in many studies. Here, indicate whether each material, system or method listed is relevant to your study. If you are not sure if a list item applies to your research, read the appropriate section before selecting a response.

Materials & experimental systems

n/a	Involved in the study	
x		Antibodies
x		Eukaryotic cell lines
x		Palaeontology and archaeology
x		Animals and other organisms
	x	Human research participants
x		Clinical data
x		Dual use research of concern

Methods

n/a	Involved in the study	
x		ChIP-seq
x		Flow cytometry
	x	MRI-based neuroimaging

Human research participants

Policy information about studies involving human research participants

Population characteristics	Discovery participants were recruited from the UK Biobank, a population-based cohort of over 500,000 individuals aged 39–73 years from 22 centers across the United Kingdom between 2006 and 2010. Our study focused on a subset of N = 22,459 (11173M/11286F) participants for each of whom complete genotype and multimodal MRI data were available for download. Subjects that have any ICD-10 coded neurological or psychiatric diseases, congenital neurological diseases, that reported themselves that they were told to have a specific neurological/psychiatric disease (which may or may not have been ICD-coded), non-Caucasian, with incomplete MRI data were excluded.
Recruitment	Discovery participants were recruited from the UK Biobank, a population-based cohort of over 500,000 individuals aged 39–73 years from 22 centers across the United Kingdom between 2006 and 2010. Our study focused on a subset of N = 22,459 participants for each of whom complete genotype and multimodal MRI data were available for download. Subjects that have any ICD-10 coded neurological or psychiatric diseases, congenital neurological diseases, that reported themselves that they were told to have a specific neurological/psychiatric disease (which may or may not have been ICD-coded), non-Caucasian, with incomplete MRI data were excluded.
Ethics oversight	Ethical approval was obtained from the Human Biology Research Ethics Committee, University of Cambridge (Cambridge, UK). Informed consent was provided by all participants (https://biobank.ctsu.ox.ac.uk/crystal/field.cgi?id=200). The UKB cohort formed the basis of our analyses. This study is under Application ID 34175: Identify biomarkers for distinguishing different mental disorders using brain images and their associations with genetic risk.

Note that full information on the approval of the study protocol must also be provided in the manuscript.

Magnetic resonance imaging

Experimental design

Design type	Resting state fMRI and sMRI were used.
Design specifications	NO task fMRI were included in our current study.
Behavioral performance measures	NO task fMRI were included in our current study.

Acquisition

Imaging type(s)	functional and structural MRI.
Field strength	3T
Sequence & imaging parameters	fMRI: The UKB dataset was collected in three sites (Manchester, Newcastle and Reading) using a standard EPI sequence, including Siemens 3-Tesla Siemens scanner (TR/TE = 735/39 ms, voxel spacing size = 2.4 × 2.4 × 2.4 mm, FOV = 88 × 88 ×64 matrix). GE-EPI with ×8 multi-slice acceleration, no iPAT, flip angle 52o, fat saturation.<br>sMRI: High-resolution anatomical MR images were acquired, including a three-dimensional T1-weighted magnetization prepared gradient echo sequence (MPRAGE) based on the Alzheimer's disease Neuroimaging Initiative protocol. The resolution of structural image is 1 × 1 × 1 mm (TR = 735 msec, TE = 39 msec, FOV: 208 × 256 × 256 matrix) and required 5 minutes for acquisition.
Area of acquisition	Whole brain analysis.

Diffusion MRI ☐ Used ☒ Not used

Preprocessing

Preprocessing software	SPM12
Normalization	Non-linear
Normalization template	EPI template
Noise and artifact removal	nuisance covariates (6 head motions + cerebrospinal fluid [CSF] + white matter [WM]) + global signal were regressed out via a general linear model from the voxel time series
Volume censoring	For the sMRI data was normalized to MNI space using the unified segmentation method in SPM12, resliced to 3 × 3 × 3 mm, and segmented into gray matter (GM), white matter (WM), and cerebral spinal fluid (CSF) using modulated normalization algorithms, resulting outputs as gray matter volume (GMV). Then the GMV were smoothed using a Gaussian kernel with a full width at half maximum (FWHM) = 6 mm. Subject outlier detection was further performed using a spatial Pearson correlation with the template image to ensure that all subjects were properly segmented.

Statistical modeling & inference

Model type and settings: Multivariate and predictive

Effect(s) tested: No task fMRI were included in this study.

Specify type of analysis: [x] Whole brain [ ] ROI-based [ ] Both

Statistic type for inference (See Eklund et al. 2016): Whole brain voxel wise analysis.

Correction: FDR

Models & analysis

n/a	Involved in the study
[x]	Functional and/or effective connectivity
[x]	Graph analysis
[ ]	[x] Multivariate modeling or predictive analysis

Multivariate modeling and predictive analysis

The study design of developing, testing and validating of the PRS-associated multimodal biomarkers is as following. Firstly, Schizophrenia PRS (pruned at r^2 < 0.1) was used as a reference to guide fALFF+GMV fusion53,61,62 to identify PRS-associated multimodal pattern in healthy Caucasian in UKB. Then the same fusion with PRS analysis was performed on healthy Caucasian (N=22,495), healthy subjects (N=24,773), and all the available (pass MRI quality control) subjects in UKB (N=37,347) with PRS pruned at r^2 < 0.1 and r^2 < 0.2, respectively, to validate the replication of the identified PRS-associated pattern within UKB. Then the identified PRS-associated multimodal components were separated as positive (Z>0) and negative (Z<0) brain regions based on the Z-scored brain maps. 2 brain masks for each of the 2 modalities were obtained, each modality has both positive and negative brain networks (4 brain imaging networks, Fig. 1b). These masks were then used as ROIs to extract features from every subject for each modality50. The mean of the voxels within the obtained ROI was calculated for each subject, generating a N_subj×4 feature vector for the 2 modalities. For non-UKB cohorts, we directly apply the brain ROIs identified from UKB to patients' rs-fMRI and sMRI to generate the 4-dimension features.
In the prediction analysis, each of the 4 vectors was normalized to mean = 0, std = 1 to avoid contribution bias in prediction. These features were treated as the linear regressors, and the symptom/cognitive scores were treated as the targeted measures. Multiple linear regression models (Eq.1) were applied to predict cognition and symptoms for SZ across 3 independent cohorts, including BSNIP-1, fBIRN and COBRE (MPRC was not included in the prediction analysis since the related clinical measures are not available). Pearson correlations between the true and predicted values were assessed. The generalization and predictability of PRS-associated brain features can be validated.

Predicted scores=β_0+fALFF_positive×β_1+fALFF_negative×β_2+GMV_positive×β_3+GMV_negative×β_4 (1)
